# A Comprehensive Analysis of Fibroblast Growth Factor Receptor 2b Signaling on Epithelial Tip Progenitor Cells During Early Mouse Lung Branching Morphogenesis

**DOI:** 10.3389/fgene.2018.00746

**Published:** 2019-01-23

**Authors:** Matthew R. Jones, Salma Dilai, Arun Lingampally, Cho-Ming Chao, Soula Danopoulos, Gianni Carraro, Regina Mukhametshina, Jochen Wilhelm, Eveline Baumgart-Vogt, Denise Al Alam, Chengshui Chen, Parviz Minoo, Jin San Zhang, Saverio Bellusci

**Affiliations:** ^1^Department of Pulmonary and Critical Care Medicine, The First Affiliated Hospital of Wenzhou Medical University, Wenzhou, Zhejiang, China; ^2^Department of Internal Medicine II, Member of the German Lung Center, Excellence Cluster Cardio-Pulmonary Systems, University of Giessen Lung Center, Giessen, Germany; ^3^Developmental Biology and Regenerative Medicine Program, Saban Research Institute of Children's Hospital Los Angeles and University of Southern California, Los Angeles, CA, United States; ^4^Department of Medicine, Cedars-Sinai Medical Center, Lung and Regenerative Medicine Institutes, Los Angeles, CA, United States; ^5^Institute of Fundamental Medicine and Biology, Kazan Federal University, Kazan, Russia; ^6^Division of Newborn Medicine, Department of Pediatrics, Children's Hospital Los Angeles, University of Southern California, Los Angeles, CA, United States; ^7^Institute of Life Sciences, Wenzhou University, Zhejiang, China; ^8^International Collaborative Research Center on Growth Factors, Wenzhou Medical University, Zhejiang, China

**Keywords:** FGF10, FGFR2b, lung, branching morphogenesis, differentiation, ß-catenin

## Abstract

This study demonstrates that FGF10/FGFR2b signaling on distal epithelial progenitor cells, via ß-catenin/EP300, controls, through a comprehensive set of developmental genes, morphogenesis, and differentiation. Fibroblast growth factor (FGF) 10 signaling through FGF receptor 2b (FGFR2b) is mandatory during early lung development as the deletion of either the ligand or the receptor leads to lung agenesis. However, this drastic phenotype previously hampered characterization of the primary biological activities, immediate downstream targets and mechanisms of action. Through the use of a dominant negative transgenic mouse model (*Rosa26rtTA; tet(o)sFgfr2b*), we conditionally inhibited FGF10 signaling *in vivo* in E12.5 embryonic lungs via doxycycline IP injection to pregnant females, and *in vitro* by culturing control and experimental lungs with doxycycline. The impact on branching morphogenesis 9 h after doxycycline administration was analyzed by morphometry, fluorescence and electron microscopy. Gene arrays at 6 and 9 h following doxycycline administration were carried out. The relationship between FGF10 and ß-catenin signaling was also analyzed through *in vitro* experiments using IQ1, a pharmacological inhibitor of ß-catenin/EP300 transcriptional activity. Loss of FGF10 signaling did not impact proliferation or survival, but affected both adherens junctions (up-regulation of E-cadherin), and basement membrane organization (increased laminin). Gene arrays identified multiple direct targets of FGF10, including main transcription factors. Immunofluorescence showed a down-regulation of the distal epithelial marker SOX9 and mis-expression distally of the proximal marker SOX2. Staining for the transcriptionally-active form of ß-catenin showed a reduction in experimental vs. control lungs. *In vitro* experiments using IQ1 phenocopied the impacts of blocking FGF10. This study demonstrates that FGF10/FGFR2b signaling on distal epithelial progenitor cells via ß-catenin/EP300 controls, through a comprehensive set of developmental genes, cell adhesion, and differentiation.

## Introduction

In mice, the first morphological evidence of lung development is seen at embryonic day (E) 9.5 with the budding of the ventral foregut endoderm, forming the tracheal primordium ventrally and the esophagus dorsally. Concomitantly, distal to the tracheal primordium, two primary lung buds form, initiating the early stages of pseudoglandular development (E9.5-E12.5) [for reviews on early lung development, see (Warburton et al., 2008, 2010; El Agha and Bellusci, 2014)]. During this early stage, the lung epithelium undergoes branching morphogenesis, a semi-stereotypical, and reiterative budding process whereby a tree-like structure, the scaffold of the future conducting airway network, is formed. At the tip of each bud reside multipotent epithelial progenitor cells, which are positive for the transcription factors SOX9 and ID2. These cells either self-renew, if they remain distally, or give rise to bronchial progenitors when they exit the tip domain, subsequently acquiring SOX2 expression (Rawlins, 2008).

Branching morphogenesis and epithelial differentiation depend on poorly understood cross-talk among a number of signaling pathways, involving fibroblast growth factors (FGF), sonic hedgehog (SHH), bone morphogenic proteins (BMP), and wingless/integrase 1 (WNT) ligands (El Agha and Bellusci, 2014). For example, fibroblast growth factor 10 (FGF10), signaling via its epithelial receptor FGFR2b, is sufficient to induce branching morphogenesis of isolated lung endoderm grown in Matrigel (Bellusci et al., 1997b). Additionally, demonstrating that FGF10 signaling is necessary to control branching morphogenesis, both *Fgf10-* and *Fgfr2b*-null embryos display lung agenesis (Sekine et al., 1999; De Moerlooze et al., 2000), while *Fgf10* hypomorphic lungs display decreased ramifications (Ramasamy et al., 2007). Less is known about the regulation of distal tip multipotent epithelial stem cell maintenance and differentiation. Interestingly, *Fgf10* gain-of-function experiments prevent the differentiation of epithelial tip cells toward the bronchial progenitor lineage (Volckaert et al., 2013).

Almost twenty years after the discovery of FGF10 as a key growth factor regulating branching morphogenesis, the primary targets and biological activities controlled by FGF10 are still unclear (El Agha and Bellusci, 2014). Addressing this issue has been difficult, since loss of *Fgf10* leads to lung agenesis, therefore leaving little tissue to study; and while conditional deletions are possible, the lapse of time separating either constitutive or inducible Cre activity (in the case of a CreERT2 system) from complete gene inactivation is usually 24 to 48 h, it is difficult to distinguish between primary and secondary effects (Abler et al., 2009). Furthermore, genetic deletion of *Fgf10* does not necessarily mean simultaneous loss of corresponding functional protein. The stability of the protein depends, for example, on the degradation rate of FGF10 present in the extracellular matrix and bound to heparin sulfate proteoglycans (Makarenkova et al., 2009; Patel et al., 2017), and it might take hours or days before a complete loss of function is achieved.

Genetically modified mouse strains, based on the reverse tetracycline transactivator (rtTA) system, do exist to conditionally inhibit FGF ligand activity at the protein level, and employ a dominant negative soluble form of FGFR2b. Soluble FGFR2b is a hybrid protein, where the extracellular part of the receptor, responsible for binding FGF ligands, is fused to the heavy chain of mouse immunoglobulin (Celli et al., 1998). This hybrid protein, once secreted, sequesters all FGFR2b ligands in the extracellular matrix.

Little research has explicitly used soluble FGFR2b to study the role of FGF signaling on early lung development, while the papers that do exist only touch on the question tangentially. For example, Hokuto et al. (2003) induced soluble FGFR2b at various pre- and postnatal stages to elicit the role played by FGF signaling in alveologenesis. While the authors reported a clear phenotype in early lungs (for example, branching defects) associated with FGF inhibition, detailed analyses of these defects were missing. Therefore, in this paper, we focused our study at E12.5, a stage where FGF10 is the only FGFR2b ligand significantly expressed. We performed both *in vitro* and *in vivo* experiments to analyze the impact of blocking FGF10 activity at the protein level. We used the developing lung as a model system to decipher the primary role of FGF10 in branching morphogenesis. Through the use of a previously validated dominant negative transgenic mouse model (*Rosa26rtTA; tet(o)sFgfr2b*; Parsa et al., 2008, 2010; Volckaert et al., 2011, 2017) we conditionally inhibited FGF10 signaling *in vivo* in E12.5 embryonic lungs via doxycycline IP injection to pregnant females, and *in vitro* by culturing lungs with doxycycline. The impact on branching morphogenesis 9 h after doxycycline administration was analyzed by morphometry, fluorescence, and electron microscopy. Gene arrays at 6 and 9 h following doxycycline administration were performed. The relationship between FGF10 and ß-catenin signaling was also analyzed using IQ1, a pharmacological inhibitor of ß-catenin/EP300 transcriptional activity.

This study demonstrates that FGF10/FGFR2b signaling on distal epithelial progenitor cells via ß-catenin/EP300 controls, through a comprehensive set of developmental genes, cell adhesion, and differentiation. Altogether, our results clarify the role of FGF10 on tip epithelial progenitor cells during development. Our transcriptomic approach has also provided a valuable dataset for future mechanistic studies aiming to characterize the role of newly found players in FGF10 signaling. Such knowledge will be instrumental to better understanding the role of FGF10 signaling at later stages of lung development, as well as during the repair process after injury.

## Results

### Expression of *Fgf* Genes Encoding the Main FGFR2b Ligands During Early Lung Development and Validation of the Transgenic Approach to Inactivate FGFR2b Ligands

**Figure 1:**
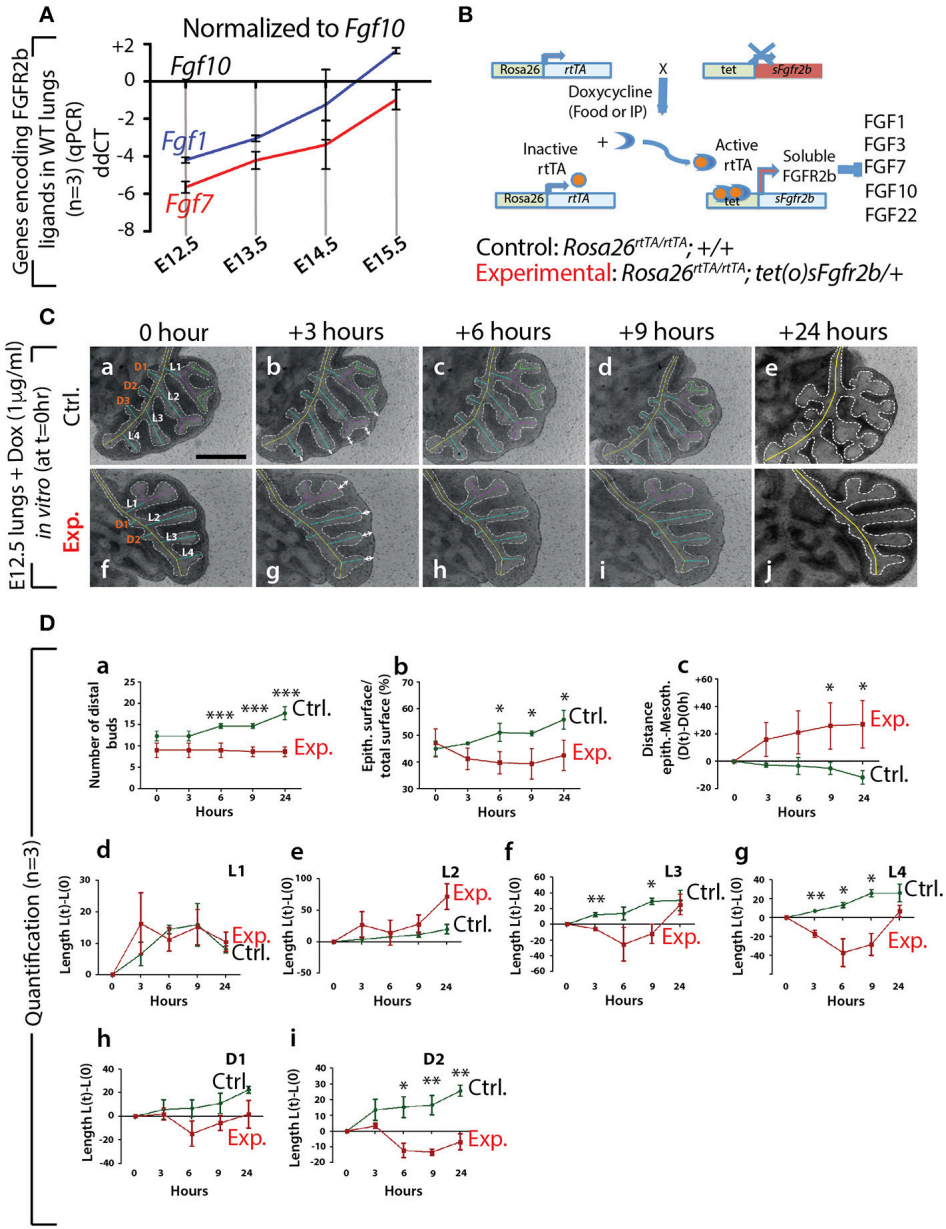
Expression of genes encoding the main FGFR2b ligands during early lung development and impact of FGFR2b ligand inactivation on branching morphogenesis **(A)** qPCR for *Fgf1, 7*, and *10* in mouse embryonic lungs at E12.5, E13.5, E14.5, and E15.5. *Fgf10* is the main ligand expressed at E12.5 (*n* = 3). **(B)** *Rosa26*^*rtTA*/*rtTA*^*; tet(O)sFgfr2b/*+ double transgenic system inducing, upon doxycycline exposure (via food, water, or IP), the expression of a soluble form of FGFR2b acting as a dominant negative receptor. **(C)** Impact of FGFR2b ligand inactivation on the branching process. Branches (a and f) are labeled according to established domain branching nomenclature (L,lateral branch; D,dorsal branch). Arrows (b and g) indicate the distance between distal tip epithelium and the adjacent mesothelium. *Scale bar:* 400 μm. **(D)** Quantification of the branching defects. (Data are presented as mean ± SEM; significance determined by unpaired two-tailed Student's *t*-test; *n* = 3; ^*^p-value < 0.05, ^**^p-value < 0.01, ^***^p-value < 0.001).

First, at different stages during embryonic lung development, we monitored by qPCR the expression of *Fgf* genes encoding ligands of FGFR2b, of which only *Fgf1, 7*, and *10* were detected (*n* = 3; Figure 1A). At E12.5, *Fgf10* was the predominantly expressed *Fgf* gene, while the expressions of *Fgf1* and *7* progressively increased during development, as previously described (Bellusci et al., 1997b). Next, we validated the double transgenic approach to block the activity of all FGFR2b ligands via the inducible expression of soluble FGFR2b using the *Rosa26*^*rtTA*/*rtTA*^*; tet(o)sFgfr2b/*+ transgenic mouse line (Figure 1B). The ubiquitous expression of soluble FGFR2b is achieved upon exposure to doxycycline (Dox), delivered via food, water, or intraperitoneal (IP) injection. Efficient inhibition of FGF10 activity occurs shortly after the soluble receptor is produced (usually within minutes after exposure to Dox; Danopoulos et al., 2013). Figure S1A shows the *in vivo* validation of this approach on lung development. Our results indicate severely impaired branching of the lung, with long, non-ramified epithelial tubes reminiscent of the primary bronchi. As expected, the earliest time points of treatment were the ones leading to the more severe phenotype. Dox-food exposure from E9.5 onwards led to complete lung agenesis (data not shown), similar to the genetic loss of *Fgf10* or *Fgfr2b* (Sekine et al., 1999; De Moerlooze et al., 2000). Figure S1B shows the *in vitro* validation of our transgenic approach. While control lungs underwent significant branching over time (Figure S1Ba,c,e,g), experimental lungs failed to branch, but instead formed long tubular extensions (Figure S1Bb,d,f,h) similar to that observed *in vivo*.

Therefore, our results indicate that *Fgf10* is the predominantly expressed FGFR2b ligand at E12.5 and that we have a validated transgenic system allowing the inducible blockade at the protein level of FGFR2b ligands. Due to our choice of E12.5 to run our experiments, we therefore conclude that inhibiting FGFR2b ligands at this stage is functionally equivalent to inhibiting FGF10 activity. In addition, supporting our choice to focus this study on FGF10, *Fgf1* and *Fgf7* knock out mice are viable and do not display any respiratory defects (Guo et al., 1996; Miller et al., 2000).

### Impact of FGF10 Inhibition on Branching Morphogenesis

We performed *in vitro* live imaging experiments for up to 24 h on control and experimental E12.5 lungs cultured with Dox (*n* = 3). This enabled us to characterize the changes occurring in the branching of the epithelium. We focused on the left lobe over this 24-h period (Figure 1C). While the number of distal buds in control lungs increased (from 12.0 ± 1.2 to 17 ± 1.5), no new buds were observed in experimental lungs (Figure 1Da). We also measured the total epithelial surface vs. the total surface of the left lobes (Figure 1Db). Our results indicate a phase of retraction of the epithelial surface in experimental lungs (−12.0% ± 4.0 at 3 h, −15.0% ± 4.2 at 6 h, −16.6% ± 5.7 at 9 h, and −9.8% ± 5.7 at 24 h). At the same time-points, the epithelial surface ratio increased in the control lungs (+4.0% ± 0.7 at 3 h, +13.0% ± 3.3 at 6 h, +12.7% ± 0.9 at 9 h, and + 24.0% ± 3.5 at 24 h). Next, we measured the distance between the epithelium at the tip of the buds and the adjacent mesothelium (see white arrows Figures 1C, Dc). We observed a progressive increase in the space between the tip epithelium and the mesothelium in experimental lungs over time (+18.0% ± 12.4 at 3 h, +24.0% ± 15.7 at 6 h, 29.5% ± 16.0 at 9 h, and 31.0% ± 17.0 at 24 h), while this distance decreased in control lungs (−8.7% ± 3.7 at 3 h, −10.5% ± 4.5 at 6 h, −16% ± 3.9 at 9 h, and −36.6% ± 1.2 at 24 h). Finally, we quantified the lengths of the different epithelial domain branches over time (Figure 1Dd–i). These domain branches were named according to the previously described nomenclature (Metzger et al., 2008b). In both control and experimental lungs, L1, L2, L3, L4, D1, and D2 were clearly visible. Note that the experimental lung shown in Figure 1Cf was slightly delayed in terms of branching, compared to the lung shown in Figure 1Ca, as is often the case between lungs within a given litter. Consequently, in the control lung, L1 and L2 were already ramified, while only L1 was in the experimental lung. In both control and experimental lungs, L3, L4, D1, and D2 were not ramified. Our results indicate that the temporal increase in length of D1 and D2 was less in experimental lungs, compared to controls (Figure 1Dh,i). Furthermore, when comparing branches that were already ramified (such as L1 in control and experimental), there was no difference between experimental and control lungs during the time period considered (Figure 1Dd). However, caution should be exercised when comparing ramified branches with non-ramified branches (such as L2 in the control and experimental lungs shown), as there tended to be an increase in the rate of lengthening of the initially non-ramified branch, compared to the initially ramified branch. This could explain why the average L2 branch length in the experimental lungs showed a greater increase over time compared to the control branch (Figure 1De).

In conclusion, our detailed analysis reveals that subtle branching defects were already apparent 3 h after exposure to Dox. The major impact of inhibiting FGF10 activity was on the epithelium, where a complete arrest in budding, and a transient retraction of the epithelium (which correlated with an increase in the distance between the mesothelium and the distal tip epithelium), was observed.

### Inhibition of FGF10 Activity Leads to Reduction of Epithelial Bud Lumen Area Associated With Cell Rearrangements

**Figure 2:**
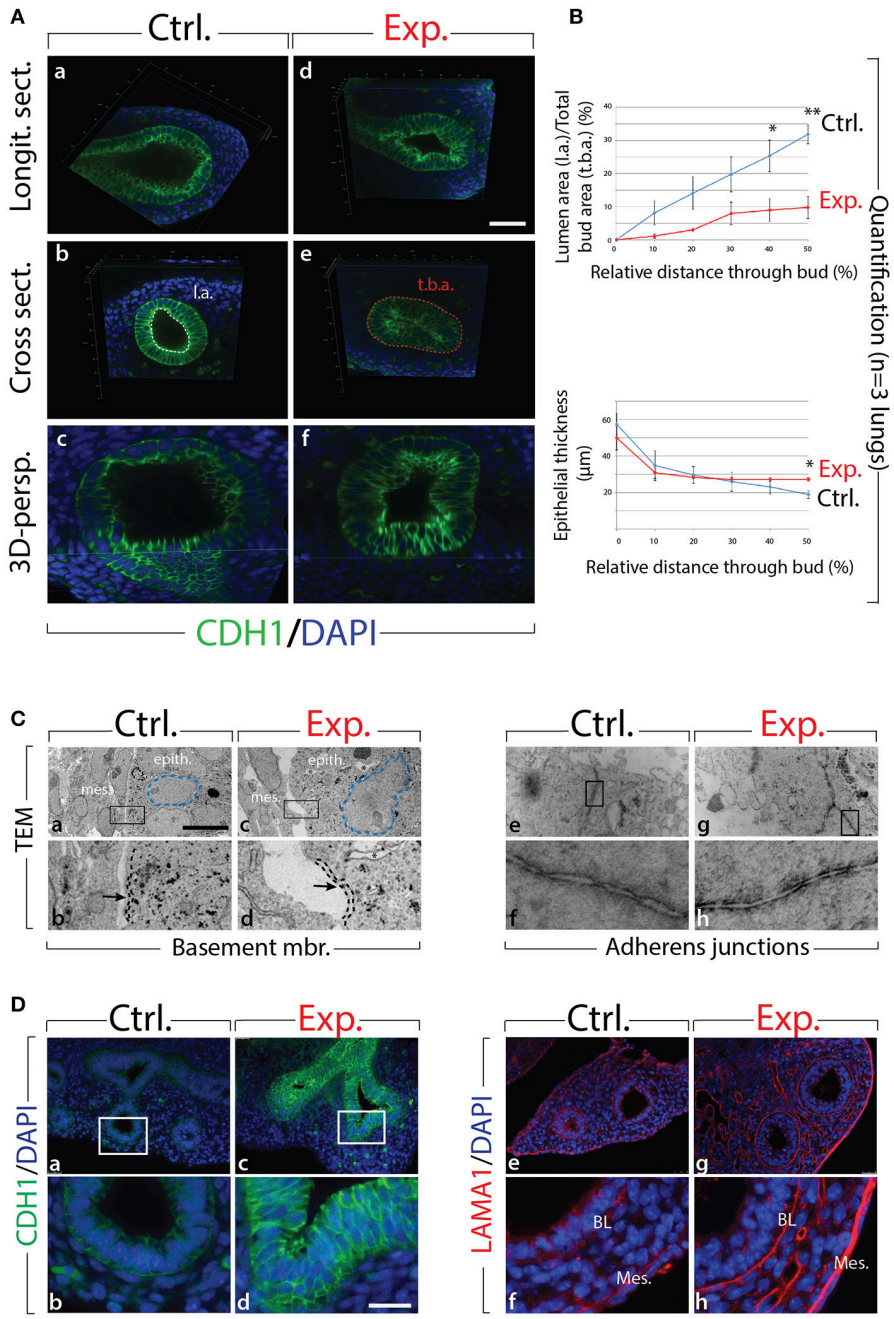
Inhibition of FGF10 activity for 9 h in E12.5 lungs leads to collapse of the epithelial bud associated with cell rearrangements and altered cell-cell adhesion **(A)** whole-mount confocal images of distal lung buds. Control buds show open lumens and an ordered epithelial monolayer (a–c), while experimental lung buds show collapsed lumens associated with multi-layered epithelium (d–f). Please note that due to our fluorescence acquisition requirements, the intensity of the signal cannot be compared between control and experimental samples. l.a. = lumen area; t.b.a. = total bud area. *Scale bar:* (a,b,d,e) 40 μm; (c,f) 16 μm. **(B)** Quantification of relative lumen area (l.a./t.b.a.) and epithelial thickness. (Data are presented as mean ± SEM; significance determined by unpaired two-tailed Student's *t*-test; *n* = 3; ^*^p-value < 0.05, ^**^p-value < 0.01). **(C)** TEM of distal lung buds highlighting the thickened basement membrane (black dashed line and arrows; a–d) and the darker staining around the adherens junctions (arrows; e–h) in experimental vs. control lungs. Note also the enlarged and irregularly shaped nuclei (blue dashed line; a,c) and the gaps between adjacent epithelial cells (asterisk; c,d) in experimental vs. control lungs. mes. = mesenchyme; epith. = epithelium. *Magnification:* (a,c) 7,750x; (e,g) 27800x; (b,d) 38,750x; (f,h) 139,000x. **(D)** Immunofluorescent staining for E-cadherin (CDH1) and laminin (LAMA1). E-cadherin shows increased expression in experimental lungs (c,d) compared to controls (a,b). Laminin deposition is increased in the basal lamina (BL) and mesothelium (Meso.) in experimental (g,h) vs. control lungs (e,f). *Scale bar:* (a,c) 75 μm; (b,d) 19 μm.

**Figure 3:**
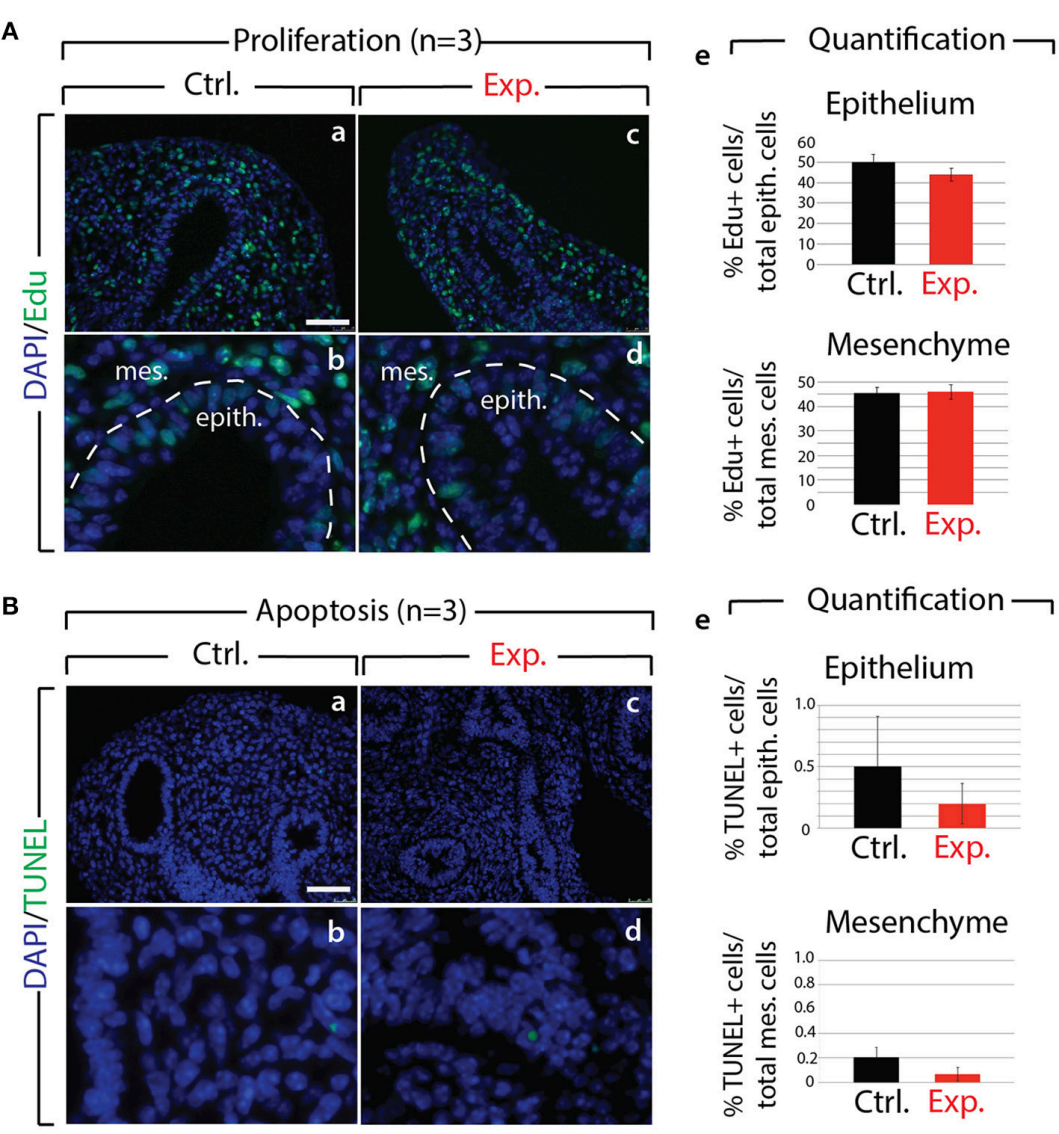
Analysis of proliferation and cell death in control and experimental lungs **(A)** Proliferation analysis following Edu injection to pregnant females in control (a,b) and experimental (c,d) E12.5 lungs. Quantification of Edu signal indicates no major difference in the number of proliferating cells in control vs. experimental lungs (e). (*n* = 3) *Scale bar:* (a,c) 50 μm; (b,d) 17 μm. **(B)** Apoptosis analysis by TUNEL showing no major difference (e) in the number of apoptotic cells in control (a,b) vs. experimental (c,d) lungs. (*n* = 3) *Scale bar:* (a,c) 50 μm; (b,d) 17 μm.

Next, we analyzed the branching defects at the cellular level using 3D-reconstructions of serial confocal images of distal epithelial buds in control and experimental lungs. These lungs were isolated 9 h following a Dox-IP to pregnant females carrying E12.5 embryos, and were whole-mount stained with CDH1 (E-cadherin) antibody. Figure 2A shows a longitudinal section, cross section, and 3D projection of control (a, c, e) and experimental (b, d, f) buds (see corresponding movies in Supplementary Materials). Quantification of the relative lumen area at different positions within the bud shows a clear reduction in this ratio in experimental buds, compared to controls (*n* = 3; Figure 2B). In addition, the average epithelial thickness was larger in experimental buds, compared to controls (Figure 2B). This increased thickness was likely a consequence of epithelial cells piling atop one another, and failing to form an ordered monolayer, as seen in control buds. Altogether, inhibition of FGF10 activity led to the collapse of the lumen within the bud, and to increased epithelial thickness, which we think are the consequences of cell rearrangements within the epithelial layer. This conclusion is supported by the fact that extensive analysis of cell proliferation and cell death in the epithelium and mesenchyme, at this time point, did not indicate any difference between control and experimental lungs (*n* = 3; Figure 3).

We also analyzed the appearance of epithelial tip cells in control and experimental lungs by transmission electron microscopy (TEM; Figure 2C). Our results reveal numerous interesting impacts of blocking FGF10 signaling in experimental vs. control epithelial tip cells, including altered Golgi morphology, decreased microvilli size and number, and opened tight junctions (see Figure S2). In terms of impacts on cell rearrangement, in experimental lungs, the thickness of the basement membrane was consistently greater than that of control lungs (see dashed line in Figure 2Cb,d). This increase in basement membrane thickness was confirmed by immunofluorescence for laminin (LAMA1; Figure 2De–h). Second, epithelial cell-cell adhesion was affected in experimental lungs, compared to controls. This is evidenced by the many large gaps between adjacent epithelial cells in experimental lungs (see the asterisks in Figure 2Cc,d), whereas adjacent epithelial cells in control samples formed tight associations with few gaps. Impacts on adhesion are further demonstrated by looking at the adherens junctions, where a darker staining was observed in experimental lungs, compared to controls (Figure 2Ce–h). This darker staining suggests an accumulation of adherens junction associated protein, most likely CDH1. Indeed, immunofluorescent staining revealed drastically increased CDH1 expression in the distal epithelium of experimental lungs vs. controls (Figure 2Da–d).

Our results demonstrate that inhibition of FGF10 signaling leads to impaired distal bud morphology, including collapsed bud lumens and thicker epithelial layers. It is likely that this phenotype is primarily a result of epithelial disorganization caused by adhesion and rearrangement defects.

### Identification of Early FGF10 Target Genes by Gene Array

**Figure 4:**
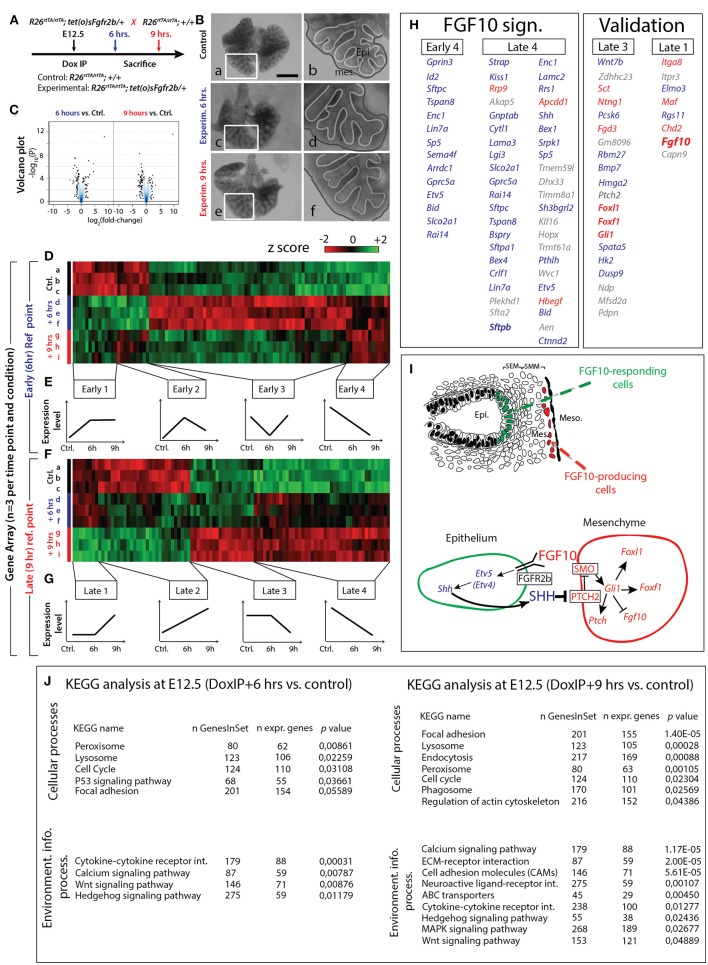
Identification of early FGF10 target genes by a gene array approach **(A)** E12.5 littermate control and experimental lungs were collected 6 or 9 h after a single Dox-IP injection to pregnant females. **(B)** FGFR2b signaling attenuation leads to visible branching defects at Dox-IP+9 h (increased mesothelial-epithelial distance and branching inhibition). Meso.=Mesothelium; Epi.=Epithelium. *Scale bar:* (a,c,e) 500 μm; (b,d,f) 175 μm. **(C)** Volcano plots showing genes which are either down- or up-regulated at 6 or 9 h Dox-IP compared to the time-matched control lungs. **(D)** Heatmap of the top 100 genes differentially expressed between experimental and control lungs at 6 hrs (based on *p*-values). **(E)** Four classes of genes, grouped according to expression pattern (Early 1–4). **(F)** Heatmap of the top 100 genes differentially expressed between experimental and control lungs at 9 h (based on *p*-values). **(G)** Four classes of genes, grouped according to expression pattern (Late 1–4). **(H)** Genes identified in the Early 4 and Late 4 clusters are direct targets of FGF10 signaling and comprise the “FGF10 gene signature.” Many genes in the Late 3 and Late 1 clusters are involved in the FGF10/SHH regulatory feedback loop (see **I**), validating the gene array approach (note the presence of *Fgf10* in the Late 1 cluster). Genes enriched in the epithelium or mesenchyme are color coded in blue or red, respectively. **(I)** Reconstitution of the FGF10-FGFR2b-ETV4/5-SHH-FGF10 signaling axis from the gene array. **(J)** KEGG results for 6 and 9 h Dox-IP vs. control. [**(D,F)** *n* = 3; see *Materials and Methods* for details on the statistical analysis of gene arrays].

Based on the previous results, we selected E12.5 as the ideal time point to determine the specific transcriptomic targets of FGF10 *in vivo* using our transgenic system. Since a single Dox-IP is sufficient to quickly induce soluble FGFR2b expression at E12.5 (Danopoulos et al., 2013), we administered single Dox-IPs to pregnant females and isolated embryos 6 or 9 h later (Figure 4A). Compared to control lungs (Figure 4Ba,b), the experimental lungs showed branching simplification and an increased distance between the distal epithelium and the mesothelium at 6 h (Figure 4Bc,d), a phenotype which was more pronounced at 9 h (Figure 4Be,f). RNA isolated from single experimental lungs at 6 and at 9 h was used for whole transcriptome analysis by gene array. As the difference in terms of size and branching between control lungs at 6 and 9 h was minimal, we chose two controls at 6 h and one control at 9 h for the gene array. Figure 4C shows the corresponding volcano plots, demonstrating significant sets of genes being either up- or down-regulated between experimental and control lungs at these two time points. Figure 4D shows the heatmap of the top 100 regulated genes (selected according to *p*-value; *n* = 3) between experimental and control lungs at 6 h, as well as the corresponding expression levels for those genes at 9 h. Figure 4F shows the heatmap of the top 100 regulated genes (selected according to *p*-value; *n* = 3) between experimental and control lungs at 9 h, as well as the corresponding expression levels for those genes at 6 h. At both the 6 and 9 h reference points, four gene clusters can be identified based on similar expression patterns (Figures 4E,G). Of great interest are the genes contained in the Early 4 and Late 4 clusters. These genes showed early down-regulation upon attenuation of FGF10 signaling, and their expression continued to decrease over time. These genes, therefore, are likely primary targets of FGF10. The heatmaps for the other gene clusters, as well as their regions of expression in the embryonic lung using the genepaint database, are shown in supplementary data (Figures S3–S10).

Figure 4H displays the list of genes found in the Early 4 and Late 4 clusters (representing the “FGF10 gene signature”), as well as Late 3 and Late 1 (presented as a validation for the array). Interestingly, Early 4 (6 h) contains only 14 genes while Late 4 (9 h) contains 43 genes (10 of which overlap with the Early 4 genes), suggesting progressive transcriptional-level changes with time. As expected, Early 4 contains genes linked to multipotent epithelial cell differentiation, such as *Sftpc* and *Id2*. The transcription factor *Etv5*, an accepted bona fide target of FGF signaling, was also among the first genes to be down-regulated. The Early 4 cluster also contains genes linked to cell adhesion (e.g., *Tspan8* and *Lin7a*), signaling and transcriptional regulation (e.g., *Gprc5a* and *Sp5*), neuronal processes (e.g., *Gprin3, Enc1*, and *Sema4f* ), apoptosis and cell cycle (e.g., *Bid* and *Rai14*), and transport (e.g., *Arrdc1* and *Slco2a1*).

The Late 4 cluster displays supplementary genes linked to epithelial differentiation, such as *Sftpa1, Sftpb*, and *Hopx*. *Etv5* was also found among these top-regulated genes. *Etv4*, a related transcription factor working redundantly with *Etv5* (Herriges et al., 2015), was also down-regulated, albeit at a lower level (data not show). Furthermore, the Late 4 cluster includes many additional genes regulating the biological processes described for the Early 4 cluster (e.g., cell adhesion genes *Lama3, Lamc2*, and *Ctnnd2*; signaling and transcriptional regulation genes *Kiss1, Akap5, Cytl1, Apcdd1*, and *Shh*; and apoptosis and cell cycle genes *Bex4, Dhx33, Klf16*, and *Aen*). Figure 4J shows KEGG pathway analyses for the 6 and 9 h time points, highlighting the cellular and signaling processes significantly regulated in experimental vs. control lungs. Significant changes in cytokine-cytokine receptor interactions, calcium signaling, WNT signaling, and Hedgehog signaling were observed at 6 h. In addition to these changes, at 9 h, we also observed significant alterations in the ECM-receptor interaction and cell adhesion molecules, reflecting the morphological alterations observed in Figure 2.

In order to gain more insight into the genes identified in each of the four groups, we made use of the online expression-profiling database “Genepaint.org” to identify the expression domain of the genes found in the different groups (Figures S3–S10). In addition, we carried out a gene array between isolated distal tip epithelial and mesenchymal cells of E12.5 wild type lungs (*n* = 3). Our results allowed us to determine the genes differentially expressed between the two compartments (see Figure S11A for details). With this data, we reanalysed the genes in the Early 4, Late 4, Late 3, and Late 1 clusters according to their relative expression in the epithelium or mesenchyme. All the genes present in the Early 4 group were more highly expressed in the epithelium, supporting the idea that at this early time point, and for this group, our global transcriptomic approach was able to identify mainly epithelial specific changes.

We then compared the differential expression of the Early 4 and Late 4 genes (FGF10 signature genes), determined from the epithelium vs. mesenchyme gene array, with their expression after FGF10 inhibition (Figures S11C,D). Genes which are expressed at high or medial levels in the epithelium, and which are highly or moderately regulated following inhibition of FGF10 activity, should be prioritized for further investigation. Promising candidates include *Lin7a, Sp5, Tspan8, Cytl1, Pthlh, Sftpa1, Sftpc*, and *Bspry* (Figure S11G).

A similar analysis was performed for the Late 3 and Late 1 groups (Figures S11E,F), which likely contain late acting, or secondary FGF10 targets. Particularly interesting from the Late 3 group is *Wnt7b*, as we have previously demonstrated that an FGF10/WNT7b loop regulates repair in conducting airways following naphthalene injury (Volckaert et al., 2011, 2017). Equally interesting in this group is the down-regulation of genes expressed preferentially in the mesenchyme and involved in Hedgehog signaling (*Foxl1, Foxf1, Gli1*, and *Ptch2*), which suggests our array captures one of the best-known epithelial-mesenchymal interactions: the FGF10-SHH interaction (reviewed in Warburton et al., 2008, 2010).

*Shh* is an epithelial gene encoding a secreted growth factor, and was down-regulated in our array concomitantly with *Etv4* and *Etv5*, beginning 6 h after FGF10 inhibition (Late 4). The combined decrease of *Etv4* and *Etv5* was likely causative for the loss of *Shh* (Herriges et al., 2015; see Discussion). Following the decrease in *Shh* expression, the mesenchymal-specific Hedgehog signaling genes *Foxl1, Foxf1, Gli1*, and *Ptch2* all showed a delayed down-regulation (only between 6 and 9 h). Furthermore, as SHH is known to regulate *Fgf10* transcription in the mesenchyme (Bellusci et al., 1997a; Lebeche et al., 1999), we also determined if *Fgf10* expression was affected by *Shh* down-regulation. We found that the Late 1 cluster, containing genes up-regulated between experimental and control at 9 h, but not at 6 h, contained *Fgf10*. Taken together, this evidence functionally validates our gene array, and leads to the model proposed in Figure 4I.

### Identification of Lung-Specific Transcription Factors Controlled by FGF10

**Figure 5:**
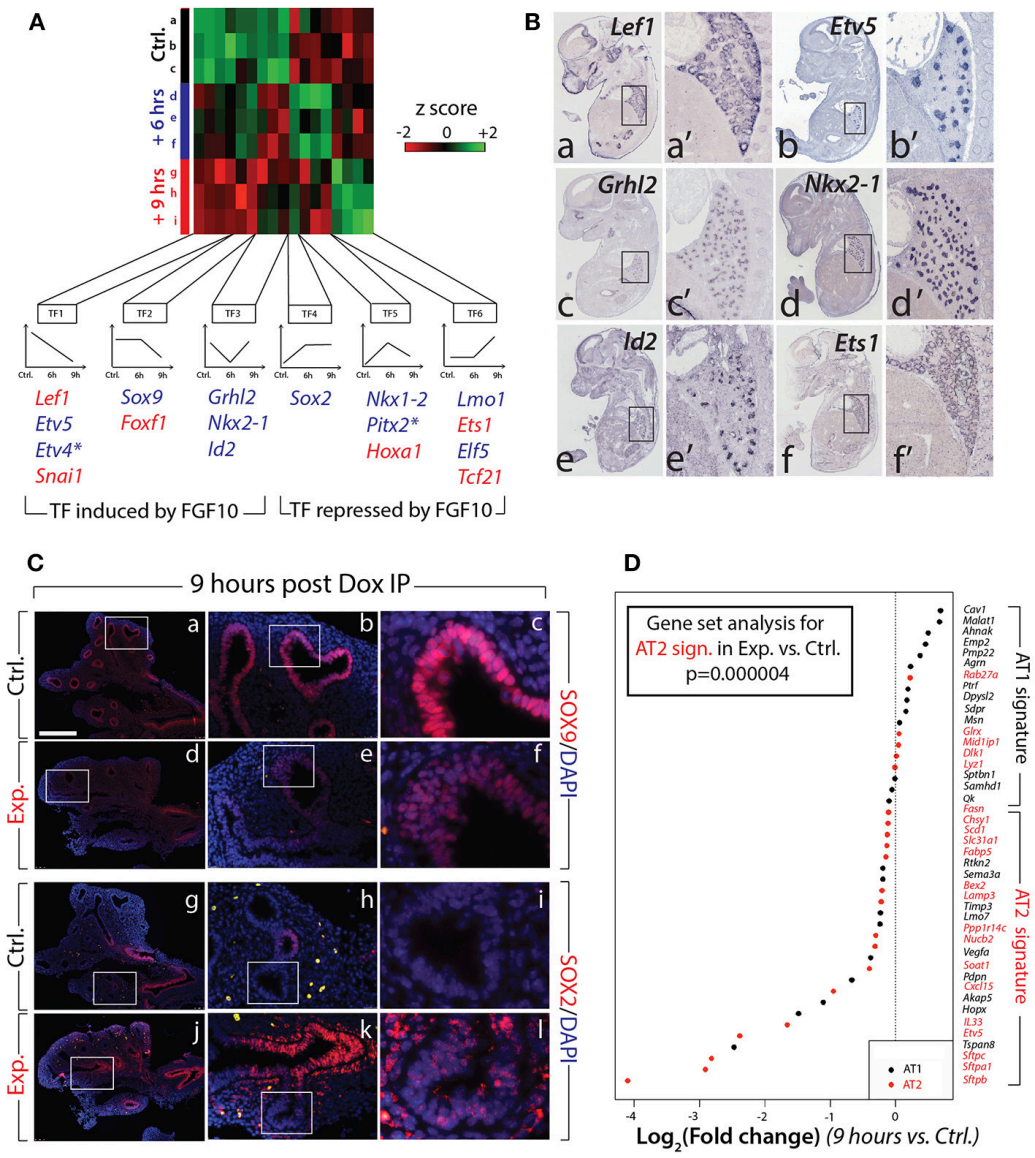
Identification of the transcription factors regulated by FGF10 and impacts on epithelial differentiation **(A)** Heatmap of highly regulated transcription factors between control, Dox-IP+6 h and Dox-IP+9 h conditions. Six classes of transcription factors (TF1-6) were identified based on their expression profiles. Genes in blue and in red are preferentially expressed in the epithelium and mesenchyme, respectively (asterisks denote genes found in both compartments). **(B)** *In situ* hybridization on E14.5 lungs for *Lef1* (a,a'), *Etv5* (b,b'), *Grhl2* (c,c'), *Nkx2-1* (d,d'), *Id2* (e,e'), and *Ets1* (f,f'). **(C)** Validation by IF of changes in SOX9 (a–f) and SOX2 (g–l) expression in experimental vs. control lungs. *Scale bar:* (a,d,g,j) 300 μm; (b,e,h,k) 100 μm; (c,f,i,l) 33 μm. **(D)** Expression of AT1 and AT2 markers in 9 h vs. control lungs. Note the significant global reduction in the expression of the AT2 markers. (*n* = 3; see *Materials and Methods* for details on the statistical analysis of gene sets).

Next, we evaluated the expression of the major transcription factors expressed in the lung at E12.5. A previous report indicated that out of 1100 transcription factors analyzed (covering 90% of all transcription factors encoded in the mouse genome), only 62 exhibited localized expression in the epithelium and/or mesenchyme of the developing lung (Herriges et al., 2012). Of these 62 genes, from our gene array, 17 were either induced or repressed with significance in experimental vs. control lungs at 6 or 9 h (*n* = 3; Figure 5A). From these 17, 11 were exclusively or predominantly expressed in the epithelium (Figure 5B shows the expression of some of these genes in the developing lung by *in situ* hybridization). Among the repressed genes in the epithelium, we found early regulation of genes such as *Etv4* and *Etv5* (belonging to the TF1 cluster), delayed regulation of *Sox9* (belonging to the TF2 cluster), as well as transiently regulated genes such as *Grhl2, Nkx2-1*, and *Id2* (from the TF3 cluster). These “repressed” genes are likely direct targets of FGF10, required to elicit the different facets of FGF10 activity. Among the induced genes in the epithelium, we found the early regulation of *Sox2* (forming the TF4 cluster), the transiently regulated genes *Nkx1–2* and *Pitx2* (from the TF5 cluster), and the late regulation of *Lmo1* and *Elf5* (from cluster TF6). These “induced” genes are likely repressed by FGF10 during early lung development.

### FGF10/FGFR2b Signaling Is Required to Maintain the Differentiation Status of the Epithelial Multipotent Progenitors

Next, we examined the impact of attenuated FGFR2b signaling on the differentiation of the multipotent epithelial progenitor cells. Figure 5C confirms the reduced expression of SOX9 distally in experimental (d–f) vs. control (a–c) lungs at 9 h post Dox-IP. In control lungs, SOX2 expression in the proximal epithelium established a clear boundary between proximal and distal regions (Figure 5Cg–i). However, in experimental lungs, SOX2 expression in the proximal epithelium expanded more distally, showing a salt and pepper expression pattern with increased expression in the mesenchyme around the conducting airway (Figure 5Cj–l).

Until recently, the close examination of epithelial tip cell differentiation was limited, as only a few signature genes denoting differentiation status were known to be expressed in those cells. However, this limitation has been overcome after a paradigm-shifting paper published by Treutlein et al. (2014), which used single cell transcriptomic approaches to characterize the genetic signatures of lung distal epithelial cells at E14.5, at E16.5, and at E18.5. The authors proposed a list of specific markers for alveolar epithelial cell type I (AT1) and type II (AT2) cells, and showed that a common progenitor cell, called a bipotent progenitor, expressed markers of both cell types. Our gene array data shown in Figure 4 indicate that some of these markers of differentiation (*Sftpc, Etv5, Sftpa1, Sftpb, Hopx, Pdpn*) are actually regulated by FGF10 in multipotent epithelial progenitors. In order to probe more extensively the status of these differentiation markers in the epithelial tip cells, we assessed the AT1 and AT2 signatures from our gene array (experimental vs. control) at the 9 h time point. Figure 5D shows a clear global reduction in the markers characteristic of the AT2 signature, with minimal change in the AT1 signature. Gene set analysis confirms a very significant difference in the AT2 signature in experimental vs. control lungs (*n* = 3; *p* = 0.000004). No significant change was observed in the AT1 signature. We therefore conclude that upon inhibition of FGF10, the tip epithelial cells, which include the progenitors for the bipotent cells, lose expression of the markers characteristic of the AT2 signature, and that globally the expression of these genes is under the control of FGF10.

### Connecting FGF10 and ß-Catenin Signaling

**Figure 6:**
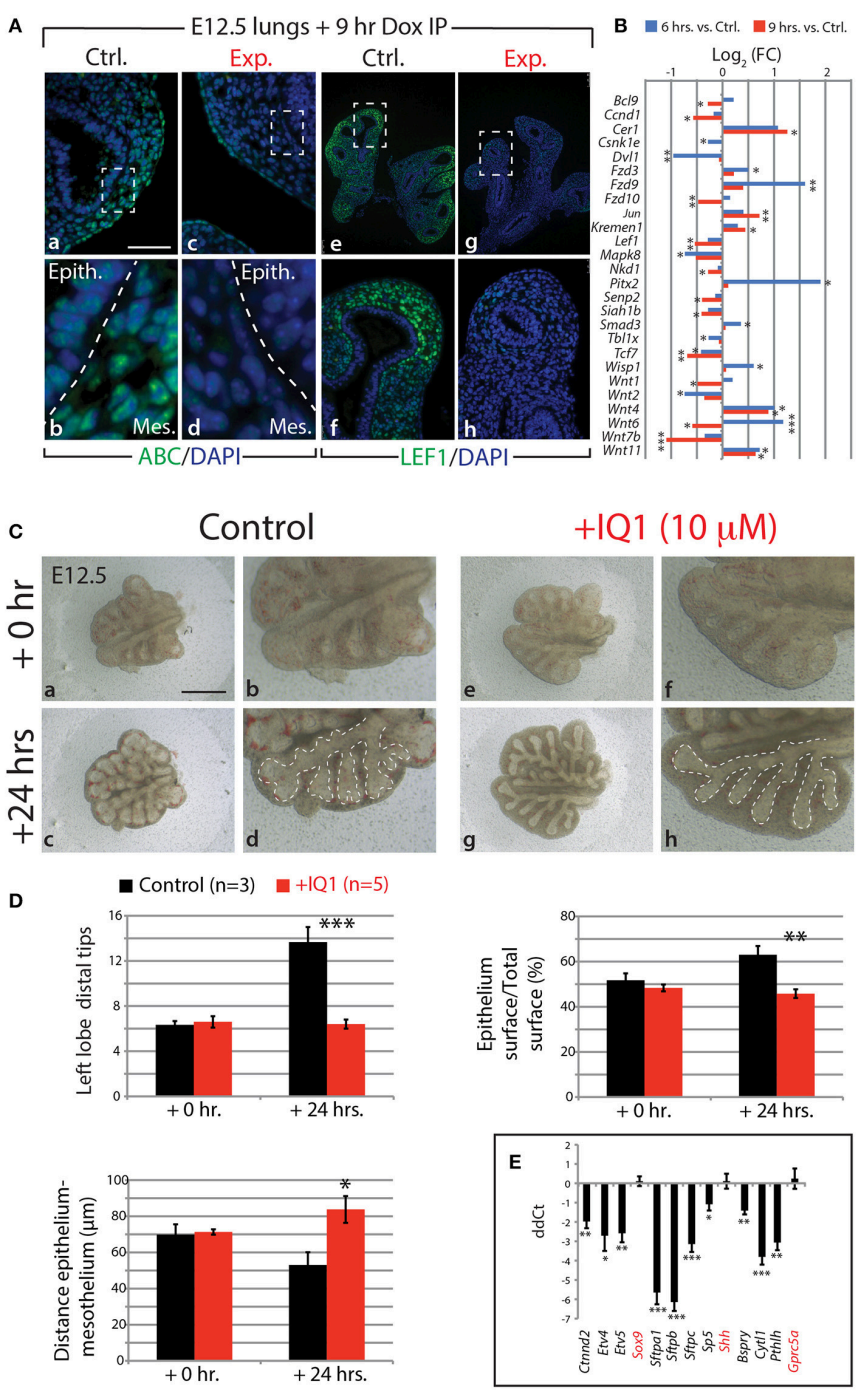
FGF10 activity is primarily mediated through ß-catenin/EP300 **(A)** IF for transcriptionally active ß-catenin (ABC) and for LEF1 in control and experimental lungs. *Scale bar:* (a,c) 50 μm; (b,d) 15 μm; (e,g) 300 μm; (f,h) 75 μm. **(B)** Gene expression changes in the canonical WNT pathway at 6 and 9 h vs. controls. (*n* = 3; see *Materials and Methods* for details on the statistical analysis of gene arrays; ^*^p-value < 0.05, ^**^p-value < 0.01, ^***^p-value < 0.001). **(C)** E12.5 lungs cultured with (experimental) or without (control) IQ1 for 24 h. Note the similarity to FGF10 inhibition on the branching in experimental lungs. *Scale bar:* (a,c,e,g) 500 μm; (b,d,f,h) 250 μm. **(D)** Quantification of branching defects in experimental vs. control lungs. **(E)** qPCR analysis on a subset of the “FGF10 gene signature.” Except for *Sox9, Shh*, and *Gprc5a*, inhibition of ß-catenin/EP300 produces a similar down-regulation of genes as in FGF10 inhibition. [**(D,E)** Data are presented as mean ± SEM; significance determined by unpaired two-tailed Student's *t*-test; *n* = 3 for control and *n* = 5 for experimental; ^*^p-value < 0.05, ^**^p-value < 0.01, ^***^p-value < 0.001].

Our KEGG analysis indicated that WNT signaling was also significantly regulated by FGF10 inhibition (Figure 4J), in particular, those genes involved in the canonical WNT pathway (*n* = 3; Figure 6B). Of note, *Wnt7b*, which codes for a growth factor secreted by the epithelium and acting on the mesenchyme, appears highly regulated by FGF10 (Figure 6B; see also Figure S11E). Downstream targets of WNT7b, such as the transcription factors *Lef1* and *Tcf7*, were also transcriptionally down-regulated. To confirm the down-regulation of WNT signaling in experimental lungs at the protein level, we examined by immunofluorescence the levels of the transcriptionally active form of CTNNB1 (ß-catenin) and the transcription factor LEF1 (Figure 6A). A loss of expression of activated ß-catenin was observed in both the epithelium and mesenchyme of experimental lungs (Figure 6Aa–d). In addition, LEF1 was also lost in the mesenchyme, correlating with the decrease in *Wnt7b* expression (Figure 6Ae–h).

Given that WNT/ß-catenin signaling is vital for proper branching morphogenesis and cellular differentiation (for a review see De Langhe and Reynolds, 2008) and that we observed a drastic reduction in activated ß-catenin in our experimental lungs, we investigated the degree to which the effect of inhibiting FGF10 was mediated through WNT/ß-catenin signaling. We made use of a pharmacological inhibitor of ß-catenin transcriptional activity, called IQ1. This inhibitor decreases the interaction between ß-catenin and one of its transcriptional coactivators, EP300, and has been shown to disrupt branching morphogenesis, and proximalize the lung epithelium, during the pseudoglandular stage of lung development (Sasaki and Kahn, 2014).

Figures 6C–E shows the results of inhibiting, *in vitro*, the ß-catenin/EP300 interaction for 24 h in E12.5 lung explants. While control lung explants displayed continued branching over this time (Figure 6Ca–d), the experimental lungs failed to branch, forming instead long epithelial tubes (Figure 6Ce–h). The failure of experimental lungs to branch, as well as the relative area of the epithelial surface and the distance between the distal epithelium and mesothelium, is quantified in Figure 6D (*n* = 3 for control and *n* = 5 for experimental). These phenotypic impacts are very similar to those seen by blocking FGF10 *in vitro* (see for comparison Figure 1Dk–m).

We also compared, by qPCR, the expression levels of a number of genes found in the “FGF10 gene signature” between experimental and control lung explants (*n* = 3 for control and *n* = 5 for experimental; Figure 6E). Ten of the 13 genes assessed showed drastic reductions in expression in IQ1 treated lung explants, similar to the down-regulation seen in our *in vivo* inhibition of FGF10. Interestingly, the transcription factor *Sox9*, as well as *Shh*, showed no change in expression between control and experimental lungs at this time point, indicating, perhaps, compensatory mechanisms.

**Figure 7:**
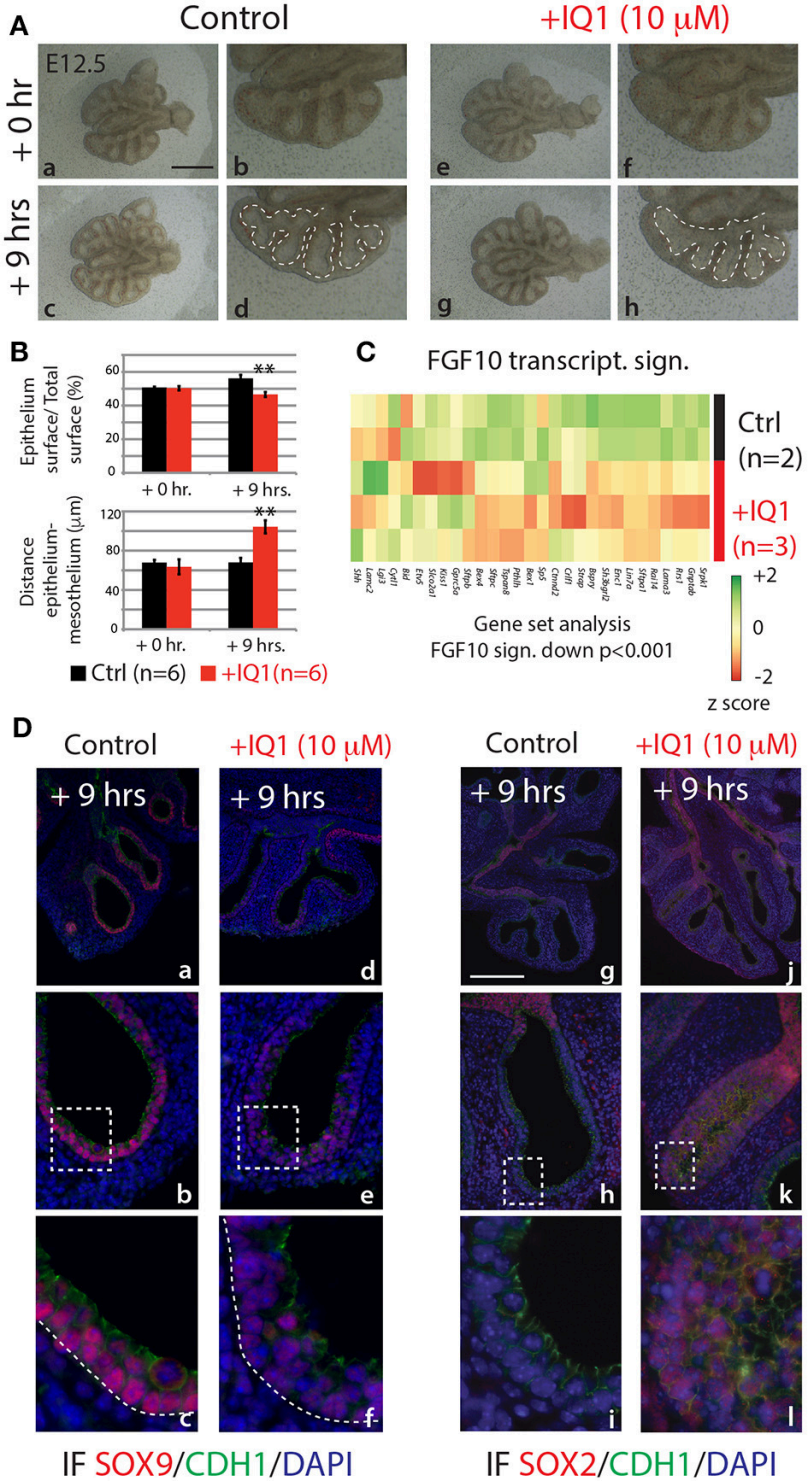
IQ1 treatment for 9 h phenocopies 9 h FGF10 inhibition **(A)** E12.5 lungs cultured with (experimental) or without (control) IQ1 for 9 h. *Scale bar:* (a,c,e,g) 500 μm; (b,d,f,h) 250 μm. **(B)** Quantification of branching defects showing a decreased relative epithelial surface and a concomitant increase in the distance between the epithelium and mesothelium. (Data are presented as mean ± SEM; significance determined by unpaired two-tailed Student's *t*-test; *n* = 6; ^**^p-value < 0.01). **(C)** A heatmap of IQ1 lungs treated for 9 h vs. control showing the “FGF10 gene signature.” A gene set analysis demonstrates the significant down-regulation of this group of genes (*n* = 2 for control and *n* = 3 for experimental; see *Materials and Methods* for details on the statistical analysis of gene sets). **(D)** IF for SOX9 (a–f) and for SOX2 (g–l). *Scale bar:* (a,d,g,j) 300 μm; (b,e,h,k) 75 μm; (c,f,i,j) 45 μm.

In the attempt to produce results more comparable to the 9 h *in vivo* inhibition of FGF10, we also conducted a 9 h inhibition of the ß-catenin/EP300 interaction (Figure 7). While the branching defects, after this time point, were less pronounced than those seen in either the 24 h IQ1 treatment, or the 9 h *in vivo* FGF10 inhibition (Figure 7Ae–h), noticeable effects on the relative epithelial surface and the distance between distal epithelium and mesothelium could be observed (*n* = 6; Figure 7B). We also conducted a gene array on control vs. IQ1 treated lung explants after 9 h of inhibition (*n* = 2 for control and *n* = 3 for experimental). A gene set analysis using the “FGF10 gene signature” revealed that this set of genes was significantly down-regulated in IQ1 treated lung explants (*p* < 0.001; Figure 7C). Finally, immunofluorescent stains for SOX9 and SOX2 protein revealed a decrease in SOX9 expression (Figure 7Dd–f), and a concomitant advancement of SOX2 expression distally (Figure 7Dj–l), reminiscent of the proximalization of lung epithelium after 9 h of FGF10 inhibition (see Figure 5C).

In summary, our data suggest that a large proportion of the regulation by FGF10 signaling of epithelial branching morphogenesis and differentiation is mediated specifically through ß-catenin/EP300 transcriptional activity.

### Discussion

**Figure 8:**
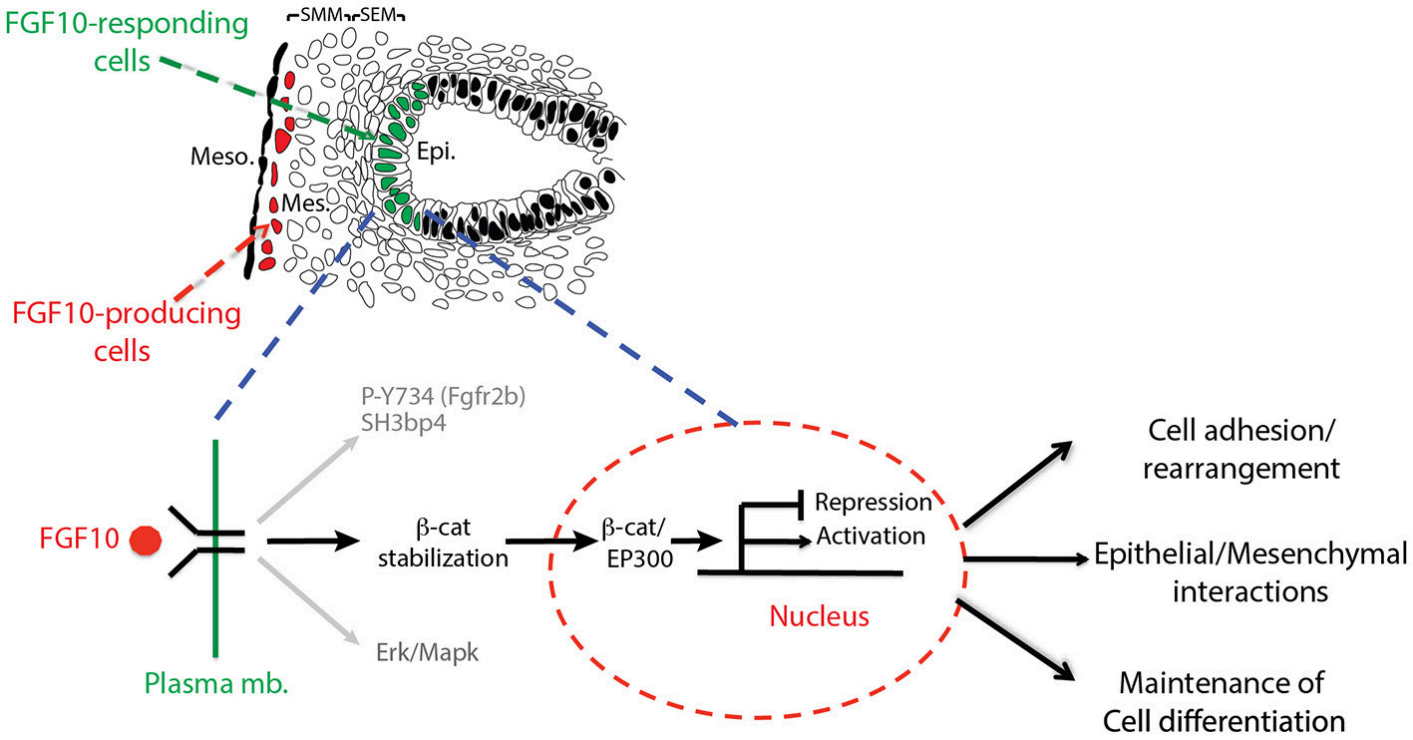
Graphical summary: FGF10 signaling leads to phosphorylation of Y734 on FGFR2 associated with Sh3bp4 recruitment (Francavilla et al., 2013). FGF10 signaling also induces Erk/Mapk phosphorylation and stabilizes ß-catenin, promoting its translocation to the nucleus where it binds to EP300, acting as a transcriptional regulator. The resulting activated (FGF10 transcriptomic signature) and/or repressed genes in turn control cell adhesion/rearrangement, contribute to epithelial/mesenchymal interactions, and maintain distal tip differentiation.

In this paper, we report the impacts on the epithelial tip cells of E12.5 lungs by blocking FGFR2b ligands, primarily FGF10. Both *in vivo* and *in vitro* experiments inhibiting FGF10 resulted in arrested epithelial branching and collapsed distal bud lumens associated with abnormal cellular adhesion. Gene arrays at 6 and 9 h inhibition revealed the transcriptomic regulation of FGFR2b signaling. From these arrays, we identified an FGF10 gene signature primarily composed of genes enriched in the epithelium and positively regulated by FGF10. We also highlighted a set of lung-specific transcription factors significantly regulated by FGF10. Our data on SOX9 and SOX2 expression, as well as gene-set analyses on differentiation markers of AT2 cells, which are found to be expressed in multipotent epithelial progenitor cells, demonstrated the proximalization of the tip epithelium and a loss of distal differentiation markers 9 h after FGF10 inhibition. Finally, the effects of blocking FGF10 signaling on ß-catenin activity were assessed. *In vitro* experiments using IQ1, a pharmacological inhibitor of the ß-catenin/EP300 interaction, were able to phenocopy a large proportion of the impacts found by FGF10 inhibition, suggesting that FGF10 signaling on E12.5 distal tip progenitors is significantly mediated via ß-catenin/EP300. These findings are summarized in a model of FGF10 signaling during pseudoglandular lung development provided in Figure 8.

### Validation, and Limitations, of *in vivo* Inhibition of FGF10 Signaling

One of the major limitations of our *in vivo* model to inhibit FGF10 signaling during pseudoglandular lung development (E12.5) is that the production of soluble FGFR2b is global, potentially inducing secondary effects. Furthermore, soluble FGFR2b is secreted into the mesenchyme, potentially inhibiting mesenchyme-specific FGF signaling. Indeed, we have recently reported that FGF10 can directly act on the rat lung mesenchyme during the late canalicular/early saccular stage (E19) to control the differentiation of lipofibroblast progenitors (Al Alam et al., 2015). Although we could not detect any significant increase in P-ERK on primary culture of lung mesenchyme isolated at the mid-pseudoglandular stage (E14.5; De Langhe et al., 2006), we cannot exclude that FGF10 is active on discrete mesenchymal subpopulations throughout the pseudoglandular stage.

To control for the potential secondary effects of our model, we validated the location of FGF10's primary targets by a gene array comparing expression of genes in the epithelium vs. mesenchyme of E12.5 wild type lungs (Figure S11), and also by the online expression-profiling database “genepaint.org” (Figures S3–S10). We are confident that our global *in vivo* approach does indeed detect the impacts of FGF10 signaling on epithelial-specific targets.

Furthermore, we assessed the well-established FGF10-SHH regulatory feedback loop during lung development as a means of validating our array (Figures 4H,I). Our array detects the down-regulation of *Shh* within 6 h of FGF10 inhibition, and also the delayed impacts on the downstream targets of SHH at 9 h, including the up-regulation of *Fgf10*. Additionally, our array supports the recently reported data showing that the inactivation of FGF-regulated *Etv4* and *Etv5* in the multipotent epithelial progenitor cells during lung development leads to the loss of *Shh* expression (Herriges et al., 2015). We therefore propose that FGF10 acts via FGFR2b, positively regulating the expression of ETV4/ETV5, and, subsequently, the SHH pathway.

### FGF10 Primary Targets

From our gene array data, we identified an “FGF10 gene signature.” These genes, primarily enriched in the epithelium, show decreased expression shortly after FGF10 inhibition, and continue to decrease during inhibition; therefore, these genes likely represent primary targets of FGF10, and are potential key mediators of FGF10/FGFR2b signaling.

We also found that FGF10 regulates many previously identified lung specific transcription factors (Herriges et al., 2012). Some of these transcription factors are established mediators of FGF10 signaling (e.g., *Etv4, Etv5, Sox9*), whereas little is known of the others in the context of FGF10 signaling. Knock-out and over-expression studies on many of these transcription factors show impacts very similar to the effects seen in our study. For example, Metzger et al. (2007, 2008a) found that *Elf5* (group TF6, Figure 5) is regulated by FGF10 and FGF7, and that over-expression of *Elf5* leads to branching defects and delayed AT2 differentiation; Quaggin et al. (1999) reported that *Tcf21* (group TF6) knock-out mice display reduced branching, smaller lungs, and a proximalization of lung epithelium at E14.5; finally, Varma et al. (2012) studied the transcription factor *Grhl2* (group TF 3, Figure 5) during lung development, and found that GRHL2 controls cell adhesion and migration, forms a positive feedback loop with NKX2-1 during branching morphogenesis, and is associated with proper AT2 differentiation.

We propose that the comprehensive set of target genes and transcription factors identified in our study is a valuable resource for future investigations on early lung branching morphogenesis and differentiation.

### FGF10′s Regulation of Tip Cell Differentiation and Morphology via SOX9

Sustained SOX9 expression in the tip epithelium of the developing lung has been associated with epithelial stem cell self-renewal. The current model predicts that individual tip cells, under the influence of FGF10, are prone to remain in the tip domain; as these cells divide, some of the daughter cells acquire bronchial progenitor characteristics associated with the exit from the tip domain.

The transcription factor SOX9 has been extensively studied during early lung development (Perl et al., 2005; Chang et al., 2013; Rockich et al., 2013). For example, Chang et al. (2013) found that knocking out *Sox9* before E12 leads to branching defects, an increase between the distal epithelium and mesothelium, and smaller lungs. Furthermore, it was found that FGFR2b signaling regulates *Sox9*, and that SOX9 suppresses the initiation of alveolar differentiation. Rockich et al. (2013) found similar findings, and also assessed the impacts on cell adhesion in *Sox9* loss-of-function E14.5 lungs. Using TEM, the authors found multiple cellular defects similar to what we found in our *in vivo* dominant negative model, including: irregularly shaped epithelial cells, gaps with protruding pseudopodia between adjacent cells, partial to complete loss of microvilli, and a disjointed basement membrane filled with gaps.

As SOX9 is lost in the distal epithelial cells of experimental lungs, the expression of SOX2 in these cells increases, further suggesting these cells are losing their multipotency, and are adopting a proximal fate. This idea is supported by the evidence, at this stage, of a loss of the AT2 signature in the multipotent progenitors upon FGF10 inhibition.

Taken together, our data suggest that the loss of SOX9, downstream of FGF10 signaling, affects the morphogenesis and multipotent potential of distal epithelial cells.

### FGF10 Regulation of ß-Catenin Levels

The importance of ß-catenin signaling during pseudoglandular branching morphogenesis has been extensively studied (reviewed in De Langhe and Reynolds, 2008). In our dominant negative *in vivo* model, transcriptionally active ß-catenin was greatly reduced in experimental lungs after 9 h inhibition (Figure 6). Since *Ctnnb1* gene expression was unaltered in our gene array, the drastic decrease in activated ß-catenin was likely a result of post-translational modifications, such as altered phosphorylation and/or reduced cytoplasmic pools of available ß-catenin.

An intriguing possibility concerning available cytoplasmic ß-catenin is the relationship between ß-catenin and CTNND2, which regulate E-cadherin stability at the adherens junction complex of epithelial cells. While ß-catenin stabilizes E-cadherin to promote cell adhesion, CTNND2 leads to E-cadherin destabilization, the release of ß-catenin to the cytoplasm, and cell motility (Lu et al., 1999; Kim et al., 2012). Indeed, high levels of stable E-cadherin have been shown to inhibit budding and branching morphogenesis in isolated embryonic lung epithelium (Liu et al., 2008). Once ß-catenin is released from E-cadherin, it contributes to the pool of transcriptionally active ß-catenin (Kam and Quaranta, 2009). With reduced CTNND2, levels of E-cadherin remain relatively high, and ß-catenin becomes sequestered at the adherens junction, being removed from the transcriptionally active pool. Thus, E-cadherin destabilization by CTNND2 regulates proper branching morphogenesis and the cytoplasmic availability of ß-catenin. Interestingly, loss of *CTNND2* in humans is associated with the “Cri du chat” syndrome (Medina et al., 2000), wherein affected babies may develop breathing problems at birth and require respiratory treatments. Additionally, a high rate of lung infection is also common during the first years of life. In the future, it will be important to delineate the impact of loss of CTNND2 on the formation and maturation of the lung epithelium in the context of homeostasis, as well as repair after injury.

Our data support the importance of the proposed CTNND2/CDH1/CTNNB1 regulatory axis. Contained in the FGF10 gene signature is the drastic reduction of *Ctnnd2* (group Late 4, Figure 4H), while E-cadherin is greatly increased in experimental lungs (Figure 2D), which could explain to an appreciable degree not only the morphological defects, but also the lack of activated ß-catenin seen in these samples.

### FGF10 Regulation via ß-Catenin/EP300

In the nucleus, ß-catenin associates with a number of transcriptional co-activators to regulate gene expression via the TCF/LEF family of DNA-bound transcription factors (reviewed in Hoppler and Kavanagh, 2007). Two of these co-activators, CBP and EP300, are critical for balancing cellular differentiation and branching morphogenesis (Miyabayashi et al., 2007; Sasaki and Kahn, 2014). Through the use of a pharmacological inhibitor of the ß-catenin/EP300 interaction, IQ1, Sasaki and Kahn (2014) report numerous impacts on pseudoglandular stage lungs, including arrested branching and an increase between the distal epithelium and adjacent mesothelium, similar to what we found in our *in vivo* inhibition of FGF10 (compare Figures 1C, 6C). Furthermore, Sasaki and Kahn (2014) show that inhibiting ß-catenin/EP300 leads to the proximalization of lung epithelium via a reduction in distal marker expression (*Bmp4* and *Fgf10*), and an increase in the proximal markers *Sox2* and *Scgb1a1*. In our 24 h IQ1 inhibition experiment, we likewise found a number of distal marker genes down regulated, including *Etv4, Etv5, Sftpa1, Sftpb*, and *Sftpc* (see Figure 6E). Interestingly, *Sox9* showed no change in expression between control and experimental lungs at this time point. In our 9 h IQ1 inhibition experiment, we were able to detect significant overall down regulation of the FGF10 gene signature, although some of the individual genes showed little regulation at this time point (see Figure 7C). This suggests that not all the genes regulated in our *in vivo* inhibition of FGF10 are similarly regulated by inhibiting ß-catenin/EP300. Furthermore, staining for SOX9 and SOX2 supports the idea that ß-catenin/EP300 sustains the multipotency of distal epithelial cells (see Figure 7D). This data is in line with a recent report showing that ß-catenin deletion in the SOX9-positive cells leads to ectopic expression of SOX2 (Ostrin et al., 2018).

In summary, the *in vitro* inhibition of ß-catenin/EP300 produces branching defects and the proximalization of epithelial cells as early as 9 h. Phenotypically, these impacts greatly overlap with the impacts of inhibiting FGF10 signaling. Many of the direct targets of FGF10 signaling are similarly regulated by ß-catenin/EP300, suggesting that FGF10 regulation is significantly mediated through ß-catenin/EP300.

In conclusion, we have carried out a comprehensive analysis of FGF10/FGFR2b signaling on epithelial tip progenitor cells during E12.5 mouse lung development. This analysis revealed a new “FGF10 transcriptomic signature” which will be instrumental in designing new mechanistic studies concerning the role of FGF10 in alveolar epithelium formation during development, as well as maintenance during homeostasis and repair after injury.

## Materials and Methods

### Contact for Reagent and Resource Sharing

Further information and requests for resources and reagents should be directed to, and will be fulfilled by, the corresponding author, Dr. Saverio Bellusci (saverio.bellusci@innere.med.uni-giessen.de).

### Experimental Model and Subject Details

#### Ethical Statement and Husbandry

Animal experiments were performed at Children's Hospital Los Angeles under the research protocols (31–08 and 31–11) approved by the Animal Research Committee and in strict accordance with the recommendations in the Guide for the Care and Use of Laboratory Animals of the National Institutes of Health. The approval identification for Children's Hospital Los Angeles is AAALAC A3276-01. Harvesting organs and tissues from wild type and mutant mice following euthanasia using pentobarbital was approved at Justus Liebig University Giessen by the federal authorities for animal research of the Regierungspraesidium Giessen, Hessen, Germany (Approved Protocol GI 20/10 Nr. G 84/2016).

All mice used to generate experimental and control embryos were housed in a specific pathogen free (SPF) environment with free access to food and water. Up to five females were housed together, while males were housed singly. Females between 9 and 12 weeks of age were used to generate embryos.

#### *In vivo* Mouse Model to Inhibit FGFR2b Ligands

*In vivo* studies were conducted using an inducible dominant negative mouse model: *Rosa26*^*rtTA*/+^*; tet(o)sFgfr2b/*+ (B6-Cg-Gt(ROSA)26Sor^tm1.1(rtTA, EGFP)Nagy^ Tg(tetO-Fgfr2b/lgh)1.3Jaw/sbel). This mouse model employs a reverse tetracycline transactivator (rtTA) under the transcriptional control of the ubiquitous *Rosa26* locus. Upon administration of doxycycline, the rtTA is able to bind to the tetracycline operator (tetO), inducing the transcription of a soluble dominant negative form of *Fgfr2b* (*sFgfr2b*). These mice were generated by crossing *Rosa26*^*rtTA*/+^ (*Gt(ROSA)26Sor*^*Tm*1.1(*rtTA, EGFP*)*Nagy*^) with *tet(o)sFgfr2b/*+ mice (Tg(tetO-Fgfr2b/Igh)1.3Jaw, obtained from Dr. Jeffrey Whitsett, Cincinnati, USA). Experimental (*Rosa26*^*rtTA*/*rtTA*^*; tet(o)sFgfr2b/*+) and littermate control (*Rosa26*^*rtTA*/*rtTA*^*;* +*/*+) embryos were generated by crossing *Rosa26*^*rtTA*/*rtTA*^*; tet(o)sFgfr2b/*+ and *Rosa26*^*rtTA*/*rtTA*^*;* +*/*+ animals.

Timed-pregnant females were used to conduct *in vivo* experiments. Doxycycline was administered either through food (625 mg doxycycline/kg food pellets), or via an intraperitoneal injection (i.p.; Dosage: 0.0015 mg doxycycline/g mouse weight) at the desired embryonic time point (E), where E0.5 was assumed to be noon on the day a vaginal copulation plug was found. At a determined time post-doxycycline i.p., a lethal dose of pentobarbital sodium was administered to animals via i.p. (Dosage: 0.4 mg pentobarbital/g mouse weight). After breathing ceased and a lack of pupil response to light was observed, cervical dislocation was performed to ensure death. Embryos were then harvested and washed in PBS with gentle shaking for ~2 min. Embryonic lungs were then dissected and prepared for subsequent analyses.

#### *In vitro* Lung Culture

Embryonic lungs used for *in vitro* experiments were obtained either from genetically modified embryos generated as described above, or from C57BL/6 wild-type embryos.

Embryonic lungs were dissected and cultured on 13 mm Whatman Track-Etch polycarbonate membranes, with 8.0 μm pores (Merck, Darmstadt, Germany) positioned atop DMEM culture medium in a 24-well culture dish [medium contained: Dulbecco's Modified Eagle Medium (1x DMEM), supplemented with D-Glucose, L-Glutamine, HEPES, Pyruvate, and Phenol red (Gibco, Paisley, UK), 10% fetal bovine serum (FBS), 1% penicillin (10,000 units/ml)-streptomycin (10 mg/ml)]. Lungs were incubated at 5% CO_2_ and 37°C for ~45 min to allow them to settle. At the beginning of the experiment (*t* = 0 h.), doxycycline dissolved in PBS (1 μg/ml) or IQ1 dissolved in DMSO (10 μM; Selleckchem, Munich, Germany) was added to the experimental lungs, while the vehicle (either PBS or DMSO) was added to control samples. Lungs were incubated at 5% CO_2_ and 37°C for the duration of the experiment.

### Method Details

#### Still and Live Image Acquisition

Brightfield images of lungs from *in vivo* and *in vitro* experiments were captured either on a Leica MZ 125 stereoscopic dissecting microscope using a Spot Insight 2.0 Mp Color Mosaic camera and Spot 4.5.9 imaging software, or were obtained from live imaging experiments using a Leica DM6000B inverted microscope, DFC 305FX camera, and Leica Application Suite Advanced Fluorescence imaging software.

#### Separation of Mesenchyme and Epithelium to Assess Relative Gene Expression in the Distal Tips Via Microarray

To assess the expression of genes in the epithelial and mesenchymal compartments of distal E12.5 lung tips, C57BL/6 wild type embryos were used. Embryonic lungs were isolated in culture medium, and the distal epithelial buds, along with the surrounding mesenchyme and mesothelium, were carefully dissected with fine-tipped pincers. The dissected tips were then immediately transferred to 500 μl undiluted dispase (Corning, Amsterdam, The Netherlands) where they were incubated for 20–30 min on ice. The dispase-digested samples were then transferred to pure FBS, and incubated for 15 min on ice, thus blocking the enzymatic activity of dispase. Using tungsten microdissection needles, the epithelium was gently separated from the surrounding mesenchyme. Separated tissues were then prepared for total RNA extraction and microarray analysis.

#### Immunofluorescence

Freshly dissected E12.5 lungs were washed in sterile PBS (2 × 5 min), fixed in 4% PFA for 20 min on ice, and then washed again (3 × 5 min). Lungs were dehydrated by successive washes in a graded ethanol series (30, 50, 70, 100, 100%) for 5 min each, and stored in 100% ethanol at −20°C.

To embed the lungs in paraffin, they were first washed in Xylol (2 × 5 min, or until clear), incubated for 1 h at 60°C in a 1:1 Xylol/paraffin mixture, washed in pure paraffin (3 × 20 min) at 60°C, and then stored in pure paraffin overnight at 60°C. Lungs were then embedded in paraffin blocks and sectioned to a thickness of 4 or 5 μm. Sections were placed in a 40°C water bath for ~30 min, and then placed on glass slides and incubated at 37°C overnight.

Before antibody staining, sections were first washed with gentle shaking in Xylol (3 × 10 min), and then in serial dilutions of ethanol (100, 70, 50, and 30%) for 3 min each, and finally in distilled water for 5 min. For each stain an antigen retrieval step was performed, which involved incubating the slides in 75–90°C citrate-based antigen unmasking solution (pH 6.0; Vector Laboratories, Peterborough, UK) for 15 min and then cooling on ice for ~30 min. Sections were then washed with PBST (1x PBS + 0.1% TWEEN20; 3 × 5 min). Blocking solution (1x PBS + 3% BSA + 0.4% TritonX) was then added atop each section for 1 h at room temperature. Primary antibodies were added to incubation buffer (1x PBS + 1.5% BSA + 0.2% TritonX) and samples were incubated overnight at 4°C (anti-SOX2, anti-Phospho-ßCatenin (Ser552), and anti-LEF1 were added at 1:100 dilution; anti-CDH1, anti-LAMA1, and anti-SOX9 were added at 1:200 dilution; see Table S1 for antibody details). Following primary antibody incubation, samples were washed in PBST (3 × 5 min) and secondary antibodies were added (all at a 1:500 dilution) for 1 h at room temperature, in the dark. Samples were washed in PBST (3 × 10 min) and PBS for 5 min, with gentle shaking. Finally, ProLong Gold antifade reagent with DAPI (Invitrogen, Schwerte, Germany) was added to each section and covered with a glass coverslip.

Sections were imaged on a Leica DM 5500B upright fluorescent microscope system, with a DFC 360FX camera, and Leica Application Suite Advanced Fluorescence imaging software. Signal intensity was optimized to either a control or experimental sample for an experiment, and the acquisition and intensity values were similarly applied to each sample in that experiment, thus ensuring valid comparisons.

#### Whole Mount Immunofluorescence and Confocal Microscopy

To assess the morphology of intact distal lung buds in control and experimental embryos, whole mount immunofluorescence followed by confocal imaging was performed.

E12.5 lungs were dissected and fixed in 4% PFA for 20 min on ice. Samples were washed in PBS + 1% TritonX (3 × 10 min), and incubated in blocking buffer (1x PBS + 1% TritonX + 10% FBS) for 1.5 h at room temperature, followed by two washes in blocking buffer. Samples were then incubated for 2 h with FITC-conjugated anti-CDH1 (Dilution: 1:200) diluted in 1/4 blocking buffer and PBS, at 4°C in the dark. Lungs were then washed in PBS (3 × 10 min), and transferred to custom made imaging dishes (composed of a 35,0/10 mm glass bottom cell culture dish (Greiner Bio-One, Frickenhausen, Germany) and a 10,0/1 mm rubber washer fixed to the middle of the dish with a suitable adhesive, thus creating an ideal well to mount and image the sample). ProLong Gold antifade reagent with DAPI was added to each well and covered with a glass coverslip.

Z-stacks of distal lung buds were obtained on a Leica TCS SP5 confocal microscopy system using Leica Application Suite X software. For each bud, the first optical section of the z-stack was acquired at the basal edge of the epithelium. Z-stack images were taken at 0.5 μm increments through the bud, until imaging was no longer possible due to complete loss of signal intensity. Compensation of intensity loss through the bud was obtained using the linear compensation by AOTF option. 3-D reconstructions and movies were created using Leica Application Suite X software.

#### Transmission Electron Microscopy

To identify the effects of FGFR2b ligand inhibition on the ultrastructure of distal epithelial and mesenchymal cells, control and experimental E12.5 lungs were prepared for transmission electron microscopy.

The chest cavities of freshly harvested E12.5 embryos were gently opened by incising from the lower abdomen, through the sternum, to just under the chin using a pair of fine dissection scissors. An incision was made along the diaphragm from the midline to the spine. The embryos were then immediately placed, ventral side up, in an immersion fixative solution containing 4% PFA + 2% sucrose + 0.05% calcium chloride + 1x PIPES buffer (0.1M, pH 7.4; Sigma-Aldrich, Taufkirchen, Germany) in a 50 ml Falcon tube, such that each sample was immersed in 5X the volume of fixative in its own tube. Tubes were placed on ice and gently shaken for 2 h, after which the fixative was removed and replaced by 4% PFA + 0.05% glutaraldehyde (GA; Sigma-Aldrich, Taufkirchen, Germany), 2% sucrose, 0.05% calcium chloride + 1X PIPES buffer (0.1 M, pH 7.4). Samples remained in this fixative overnight at 4°C.

The next morning, the samples were processed for routine transmission electron microscopy. Briefly, the fixed lungs were dissected and placed into molten agar, which was allowed to harden before the samples were cut longitudinally in half. Each half was fixed for 30 min in 1.5% GA fixative containing 2% sucrose + 0.05% calcium chloride + 1X PIPES buffer (0.1 M, pH 7.4). The fixative solution was then removed and samples were washed with 1X PIPES (3 × 5 min). Samples were then incubated for 1 h at room temperature in reduced osmium fixation solution containing 0.15% sodium hexacyanoferrate(II) and 2% reduced osmium, then washed very briefly with distilled water, and dehydrated via washes in a graded ethanol series (70, 80, 90, 100%), 3 times 10 min each step. Samples were then embedded by immersion in propylene oxide (3 × 5 min), in 1:1 propylene oxide:Agar 100 epoxy resin (1 × 30 min) following the manufacturer's instructions to produce blocks of medium hardness (Agar Scientific, Essex, UK), and finally in pure Agar 100 epoxy resin in a desiccation chamber at room temperature overnight.

The Agar 100 resin-penetrated lungs were then flat embedded into fresh Agar 100 resin and polymerized at 60°C for at least 2 days, or until complete polymerization was achieved. Ultrathin sections were then prepared and micrographs were obtained using a Zeiss LEO 906 transmission electron microscope equipped with a TRS slow-scan 2K CCD camera and ImageSP software.

#### DNA Isolation and PCR

DNA was isolated from the tails and hind limbs of E12.5 embryos. Gene-specific primers were used to detect the presence of *Rosa26rtTA* (wild type and transgene specific forward primer 5′- GAG TTC TCT GCT GCC TCC TG; wild type specific reverse primer 5′- CGA GGC GGA TAC AAG CAA TA; transgene specific reverse primer 5′- AAG ACC GCG AAG AGT TTG TC; expected product size of ~200 bp for the transgene and 322 bp for the wild type) and *tet(o)sFgfr2b* (transgene specific forward primer 5′- GAA GGA GAT CAC GGC TTC C; transgene specific reverse primer 5′- AGA CAG ATG ATA CTT CTG GGA CTG T; expected product size of 110 bp). The PCR reaction mix was calculated for 20 μl per reaction, and included 10 μl 2xTaq PCR Master Mix (Qiagen, Hilden, Germany), primers at a final concentration of 0.2 μM, RNase-free water, and up to 1 μg of genomic DNA. PCRs were performed in a C1000 Thermocycler (Bio-Rad). The cycling protocol to amplify *Rosa26rtTA* was as follows: initial denaturation at 94°C for 3 min; 35 cycles of denaturation at 94°C for 1 min, annealing at 63°C for 1 min, and extension at 72°C for 1.5 min; final extension at 72°C for 5 min; and hold at 4°C. The protocol to amplify *tet(o)sFgfr2b* was as follows: initial denaturation at 95°C for 2 min; 35 cycles of denaturation at 95°C for 5 seconds, annealing at 58°C for 5 s, and extension at 72°C for 20 s; final extension at 72°C for 2 min; and hold at 4°C. Capillary gel electrophoresis was performed using a QIAxcel Advanced capillary electrophoresis system (Qiagen).

#### RNA Isolation and RT-qPCR

Whole embryonic lungs or separated epithelium and mesenchyme used for total RNA isolation were first put in 700 μl QIAzol Lysis Reagent (Qiagen, Hilden, Germany). For tissue disruption and homogenization, the samples were transferred to gentleMACS M Tubes and homogenized in a gentleMACS Dissociator (Miltenyi Biotec) for 1 min. Total RNA was then isolated using the miRNeasy Mini Kit (Qiagen, Hilden, Germany), and eluted in 30 μl RNase-free water. RNA amount and purity was assessed with a NanoDrop 2000c (Thermo Scientific). Up to 1 μg of total RNA for each sample was then reverse transcribed using the QuantiTect Reverse Transcription Kit (Qiagen, Hilden, Germany).

Primers were designed to amplify specific mature mRNAs using NCBI's primer-BLAST option (https://www.ncbi.nlm.nih.gov/tools/primer-blast/; last accessed, 01–08–2018). Primers were further validated by PCR-based gel electrophoresis (see Table S2 for primer sequences). qPCR reaction mixtures were set up using the PowerUp SYBR Green Master Mix (Thermo Fisher, Schwerte, Germany), with a final volume of 20 μl for each reaction. Reaction mixtures included the following components: 10 μl of 2X PowerUp SYBR Green Master Mix, between 300 and 800 nM of each primer, between 1 and 10 ng cDNA, and nuclease-free water. Samples were run with two or three technical replicates on a LightCycler 480II (Celli et al.) using the following protocol: UDG activation at 50°C for 2 min; DNA polymerase activation at 95°C for 2 min; and 40 cycles of denaturation at 95°C for 15 s, annealing at 60°C for 15 s, and extension at 72°C for 1 min. To validate amplification specificity, a dissociation step was also included for each sample. Threshold cycles (Ct) were calculated and used for relative expression analyses, using mouse *Hprt* as the reference gene.

#### Microarray Analysis

Differential gene expression was investigated using microarray analysis. Depending on the amount of RNA isolated per sample in an experiment, one of two possible microarray protocols was used. For RNA concentrations >50 ng/μl, the T7-protocol was followed. In this protocol, purified total RNA was amplified and Cy3-labeled using the LIRAK kit (Agilent Technologies, Waldbronn, Germany) following the kit instructions. Per reaction, 200 ng of total RNA was used. The Cy3-labeled aRNA was hybridized overnight to 8 × 60 K 60mer oligonucleotide spotted microarray slides (Agilent Technologies, design ID 028005).

For experiments where samples yielded <50 ng/μl of RNA, the SPIA-protocol was utilized. In this protocol, purified total RNA was amplified using the Ovation PicoSL WTA System V2 kit (NuGEN Technologies, Leek, The Netherlands). Per sample, 2 μg amplified cDNA was Cy-labeled using the SureTag DNA labeling kit (Agilent Technologies). The Cy3-labeled aRNA was hybridized overnight to 8 × 60 K 60mer oligonucleotide spotted microarray slides (Agilent Technologies, design ID 074809).

For each protocol, hybridization, and subsequent washing and drying of the slides were performed following the Agilent hybridization protocol. The dried slides were scanned at 2 μm/pixel resolution using the InnoScan is900 (Innopsys). Image analysis was performed with Mapix 6.5.0 software, and calculated values for all spots were saved as GenePix results files. Stored data were evaluated using the R software (version 3.3.2) and the limma package (version 3.30.13) from BioConductor. Gene annotation was supplemented by NCBI gene IDs via biomaRt (last accessed 08–03–2018).

#### Assessing Proliferation and Apoptosis

Proliferation in E12.5 lungs was assessed using the Click-iT EdU Imaging Kit (Invitrogen, Schwerte, Germany). 5-ethynyl-2′-deoxyuridine (EdU), a nucleoside analog of thymidine incorporated into DNA during DNA synthesis, was injected (i.p.) 2 h before pregnant females were sacrificed (Dosage: 0.005 mg EdU / g mouse weight). Embryonic lungs were harvested, paraffin embedded, sectioned and placed on glass slides. Sections were then deparaffinised and stained for EdU according to the manufacturer's protocol.

Apoptosis was assessed using the TdT-mediated dUTP Nick-End Labeling (TUNEL) assay. The assay was performed using the DeadEnd Fluorometric TUNEL System (Promega, Mannheim, Germany). E12.5 lungs were harvested, paraffin embedded, sectioned and placed on glass slides. The assay was performed according to the manufacturer's protocol.

#### Gene Expression Patterns

To assess the expression patterns of genes in early stage embryonic lungs (E14.5), the online database genepaint.org was used (last accessed 01–08–2018). Each of the genes significantly regulated in our *in vivo* studies was entered in genepaint. The whole embryo section displaying the clearest gene expression in the lung, along with a magnification of the lung itself, was chosen for the figures.

### Quantification and Statistical Analysis

#### Morphometric Quantifications

Using still images of lungs, mesothelium and airways were traced in Adobe Illustrator CS6 (version 16.0.4) to create skeletal outlines. These outlines were exported and lengths and areas were quantified either using MetaMorph (version 1.5.0) or FIJI (version 2.0.0-rc-68/1.52g) software.

Significance was determined by unpaired two-tailed Student's *t*-tests. All data are presented as mean ± SEM. Values of *p* < 0.05 were considered significant. The number of independent samples (*n*) can be found in the figures.

#### Relative Gene Expression From qPCR Data

ΔCt and ΔΔCt values were calculated according to the following formulas:

(1) $$\begin{array}{r} \Delta Ct\;=Ct_{\text{Reference}}-Ct_{\text{gene of interest}} \end{array}$$

Note, this equation accounts for the fact that Ct is proportional to the –log of gene expression. ΔCt is therefore positively related to the expression of the gene of interest.

(2) $$\begin{array}{r} \Delta \Delta Ct_{\text{Experimental}-\text{control}}=Mean\Delta Ct_{\text{Experimental}}-Mean\Delta Ct_{\text{Control}} \end{array}$$

Unpaired two-tailed Student's *t*-tests were performed on the ΔCt values, which can be assumed to be normally distributed. Number of “*n*” and significance level is indicated either in the figures or in the figure legends.

#### Microarray Analyses

Mean spot signals were background corrected with an offset of 1 using the NormExp procedure on the negative control spots. The logarithms of the background-corrected values were quantile-normalized. The normalized values were then averaged for replicate spots per array. From different probes addressing the same NCBI gene ID, the probe showing the maximum average signal intensity over the samples was used in subsequent analyses. Genes were ranked for differential expression using an unpaired two-tailed Student's *t*-test on a moderated t-statistic, and heatmaps were generated displaying genes according to descending *p*-values. Gene set tests were done on the ranks of the *t*-values, using the function “geneSetTest” in the limma package from BioConductor. The number of independent samples (*n*) can be found in the figures. Gene sets were either user defined or, for pathway analyses, according to the KEGG database (last accessed 08–03–2018). The data from the microarray experiments have been deposited in the NCBI's gene expression omnibus (GEO accession GSE124157).

## Author Contributions

SB, MJ, JZ, PM, and CC: concept and design; MJ, SaD, AL, SoD, JW, GC, and RM: acquisition of data; SB, MJ, JW, EB-V, and DA: analysis and interpretation; MJ, SaD, SB, and JZ: drafting and editing of the manuscript. All authors read and approved the final manuscript.

### Conflict of Interest Statement

The authors declare that the research was conducted in the absence of any commercial or financial relationships that could be construed as a potential conflict of interest.

## Supplementary Material

Figure S1 Experimental validation of the *Rosa26*^*rtTA*/*rtTA*^*; tet(o)sFgfr2b/*+ double transgenic mice **(A)** Pregnant females carrying control (*Rosa26*^*rtTA*/*rtTA*^*;* +*/*+*)* and experimental *(Rosa26*^*rtTA*/*rtTA*^*; tet(o)sFgfr2b/*+*)* embryos were fed with Dox food starting either at E10.5, E11.5, E13.5, or E14.5 and sacrificed at E18.5. The lungs were dissected and shown in c–f. *Scale bar:* (b–f) 500 μm. **(B)** Pregnant females carrying control (a,c,e,g and a',c',e',g') and experimental (b,d,f,g and b',d',f',h') embryos were sacrificed at E12.5. The lungs were dissected and cultured for 72 h in the presence of Dox added to the culture medium. *Scale bar:* (a–h) 500 μm; (a'–h') 125 μm.

Figure S2 Transmission electron microscopy: Compared to controls, experimental lungs (DoxIP + 9 h) show reduced numbers and stunted microvilli (see black arrows, a–d), opened tight junctions (see black arrows in e and g; white asterisks in f and h), and flattened Golgi with increased staining (see black arrows, i–l). epith. = epithelium. *Magnification:* (a,c,e,g,i,k) 27,800x; (b,d,f,h,j,l) 139,000x.

Figure S3 Genes and Expression pattern found in the Early 1 cluster **(A)** Graphical representation of changes in the level of gene expression over time. **(B)** Heat map. **(C)** Corresponding *in situ* hybridization results at E14.5 from genepaint.

Figure S4 Genes and Expression pattern found in the Early 2 cluster **(A)** Graphical representation of changes in the level of gene expression over time. **(B)** Heat map. **(C)** Corresponding *in situ* hybridization results at E14.5 from genepaint.

Figure S5 Genes and Expression pattern found in the Early 3 cluster **(A)** Graphical representation of changes in the level of gene expression over time. **(B)** Heat map. **(C)** Corresponding *in situ* hybridization results at E14.5 from genepaint.

Figure S6 Genes and Expression pattern found in the Early 4 cluster **(A)** Graphical representation of changes in the level of gene expression over time. **(B)** Heat map. **(C)** Corresponding *in situ* hybridization results at E14.5 from genepaint.

Figure S7 Genes and Expression pattern found in the Late 1 cluster **(A)** Graphical representation of changes in the level of gene expression over time. **(B)** Heat map. **(C)** Corresponding *in situ* hybridization results at E14.5 from genepaint.

Figure S8 Genes and Expression pattern found in the Late 2 cluster **(A)** Graphical representation of changes in the level of gene expression over time. **(B)** Heat map. **(C)** Corresponding *in situ* hybridization results at E14.5 from genepaint.

Figure S9 Genes and Expression pattern found in the Late 3 cluster **(A)** Graphical representation of changes in the level of gene expression over time. **(B)** Heat map. **(C)** Corresponding *in situ* hybridization results at E14.5 from genepaint.

Figure S10 Genes and Expression pattern found in the Late 4 cluster **(A)** Graphical representation of changes in the level of gene expression over time. **(B)** Heat map. **(C)** Corresponding *in situ* hybridization results at E14.5 from genepaint.

Figure S11 Relative level of expression of the genes of interest in epithelium and mesenchyme of WT E12.5 lungs and regulation of gene expression upon FGF10 inhibition **(A)** Determination of genes differentially expressed in the distal epithelium vs. mesenchyme of E12.5 wild type lungs by gene array (*n* = 3; see *Materials and Methods* for details on the statistical analysis of gene arrays). **(B)** Impact of *in vivo* FGF10 inhibition on lung branching at 6 and 9 h using our double transgenic system. Corresponding gene arrays allowed the identification of genes belonging to Early 4, Late 4, Late 3, and Late 1 groups. **(C)** Analysis of the genes found in the Early 4 cluster. The first LogFC (identified with “^A^” after the gene) represents the differential expression of this gene in the epithelium vs. mesenchyme of WT E12.5 lungs. The second LogFC (identified with “^B^” after the gene) represents the level of regulation upon FGF10 inhibition. Blue indicates genes enriched in the epithelium, and red indicates genes enriched in the mesenchyme. Genes in black were not found in our gene array in **(A)**. Note that all the genes in Early 4 are blue, and therefore enriched in the epithelium. Some of these genes were differentially expressed in the epithelium at a high level (Log_2_FC more than 2; *Id2, Sftpc, Tspan8, Lin7a, Sp5, Sema4f*, *Arrdc1, Gprc5a*, and *Slco2a1*), a medial level (Log_2_FC between 1 and 2; *Etv5* and *Rai14*), or a lower level (Log_2_FC between 0 and 1; *Gprin3, Enc1*, and *Bid*). We found that some of these genes were highly regulated upon FGF10 inhibition (Log_2_FC <-2; *Gprin3*), moderately regulated (Log_2_FC between −1 and −2; *Id2, Sftpc, Tspan8, Lin7a, Sp5, Sema4f*, *Gprc5a, Etv5, Slco2a1*), or weakly regulated (Log_2_FC between 0 and −1; *Enc1, Arrdc1, Bid, Rai14*). It is likely that genes which are expressed at high or medial levels in the epithelium, and which are highly or moderately regulated following inhibition of FGF10 activity, are involved in mediating FGF10 activity. One exception is *Gprin3*, which is only weakly enriched in the epithelium, but which nonetheless is highly regulated by FGF10. **(D)** Analysis of the genes found in the Late 4 cluster. Note again that most of the genes are blue, confirming the enrichment in the Late 4 cluster for epithelial genes. Out of 43 genes, 29 were preferentially expressed in the epithelium, two were preferentially expressed in the mesenchyme, while information on the 11 other genes was not available in the array. With the exception of *Gprin3, Id2, Arrdc1*, and *Sema4f*, all the other genes found in the Early 4 cluster are also found in the Late 4 group. Some of these genes were expressed at a high level in the epithelium (Log_2_FC more than 2; *Cytl1, Lama3, Slco2a1, Gprc5a, Sftpc, Tspan8, Bspry, Sftpa1, Bex4, Crlf1, Lin7a, Shh, Bex1, Sp5, Ctnnd2*, and *Pthlh*), at a medial level (Log_2_FC between 1 and 2; *Kiss1, Lgi3, Rai14, Sftpb, Sh3bgrl2*, and *Etv5*), or at a lower level (Log_2_FC between 0 and 1; *Strap, Gnptab, Enc1, Lamc2, Rrs1, Srpk1*, and *Bid*). We also classified the genes in the Late 4 cluster according to their regulation following FGF10 inactivation: highly regulated (Log_2_FC <-2; *Cytl1, Sftpc, Bspry, Sftpa1, Lin7a, Sftpb, Sp5, Pthlh*, and *Etv5*), moderately regulated (Log_2_FC between −1 and −2; *Strap, Kiss1, Lgi3, Slco2a1, Gprc5a, Bex4, Enc1, Lamc2, Sh3bgrl2*, and *Ctnnd2*), or weakly regulated (Log_2_FC between 0 and −1; *Rrp9, Gnptab, Lama3, Rai14, Crlf1, Rrs1, Shh, Bex1, Srpk1*, and *Bid*). As with the Early 4 analysis, it would be logical to prioritize genes for future studies based both on their expression in the epithelium and their level of regulation by FGF10. *Cytl1, Sftpc, Bspry, Sftpa1, Lin7a, Sp5*, and *Pthlh* fit these prioritization criteria. **(E)** Analysis of the genes found in the Late 3 cluster. Note the presence of genes belonging to the Hedgehog signaling pathway. This group contains 20 genes, eight of which were preferentially expressed in the epithelium, five preferentially expressed in the mesenchyme, and six other genes not identified in the epithelium vs. mesenchyme array. Of the genes contained in the Late 3 cluster, some were expressed at a high level in the epithelium (Log_2_FC more than 2; *Wnt7b*, and P*csk6*), at a medial level (Log_2_FC between 1 and 2; *Bmp7*, and *Dusp9*), or at a lower level (Log_2_FC between 0 and 1; *Rbm27, Hmga2, Spata5*, and *Hk2*). We also classified the genes according to their level of regulation following FGF10 inactivation: highly regulated (Log_2_FC <-2; no genes), moderately regulated (Log_2_FC between −1 and −2; *Wnt7b, Pcsk6, Fgd3, Hmga2, Gli1, Hk2*, and *Dusp9*), or weakly regulated (Log_2_FC between 0 and −1; *Sct, Ntng1, Rbm27, Bmp7, Foxf1*, and *Spata5*). **(F)** Analysis of the genes found in Late 1 cluster. Note the presence of *Fgf10* in this cluster. **(G)** Graphical representation of the FGF10 gene signature showing those genes both enriched in the epithelium (X-axis) and down-regulated after 9 hrs FGF10 inhibition (Y-axis).

Table S1 Primary antibodies.

Table S2 Primer sequences for qRT-PCR.

Movie S1 Longitudinal section of control bud.

Movie S2 Longitudinal section of experimental bud (9 h Dox-IP).

Movie S3 Cross section of control bud.

Movie S4 Cross section of experimental bud (9 h Dox-IP).
