# Supplementary figures and images for: A Comprehensive Analysis of Fibroblast Growth Factor Receptor 2b Signaling on Epithelial Tip Progenitor Cells During Early Mouse Lung Branching Morphogenesis
Source: Front Genet. 2019 Jan 23;9:746. doi: 10.3389/fgene.2018.00746 (PMC6351499; doi:10.3389/fgene.2018.00746)

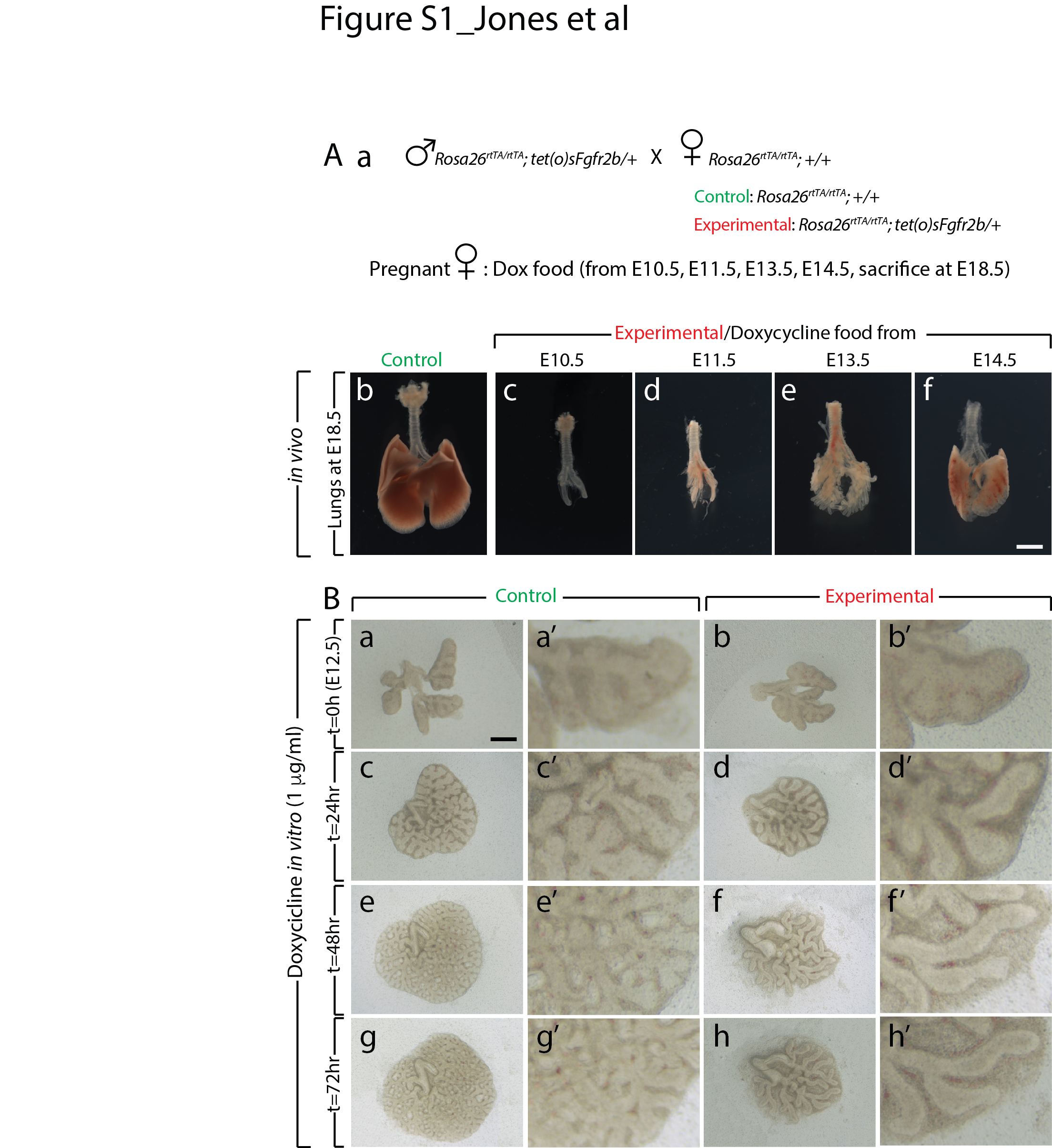

Supplement: Figure S1 — Experimental validation of the Rosa26rtTA/rtTA; tet(o)sFgfr2b/+ double transgenic mice (A) Pregnant females carrying control (Rosa26rtTA/rtTA; +/+) and experimental (Rosa26rtTA/rtTA; tet(o)sFgfr2b/+) embryos were fed with Dox food starting either at E10.5, E11.5, E13.5, or E14.5 and sacrificed at E18.5. The lungs were dissected and shown in c–f. Scale bar: (b–f) 500 μm. (B) Pregnant females carrying control (a,c,e,g and a',c',e',g') and experimental (b,d,f,g and b',d',f',h') embryos were sacrificed at E12.5. The lungs were dissected and cultured for 72 h in the presence of Dox added to the culture medium. Scale bar: (a–h) 500 μm; (a'–h') 125 μm. [file Image_1.JPEG]

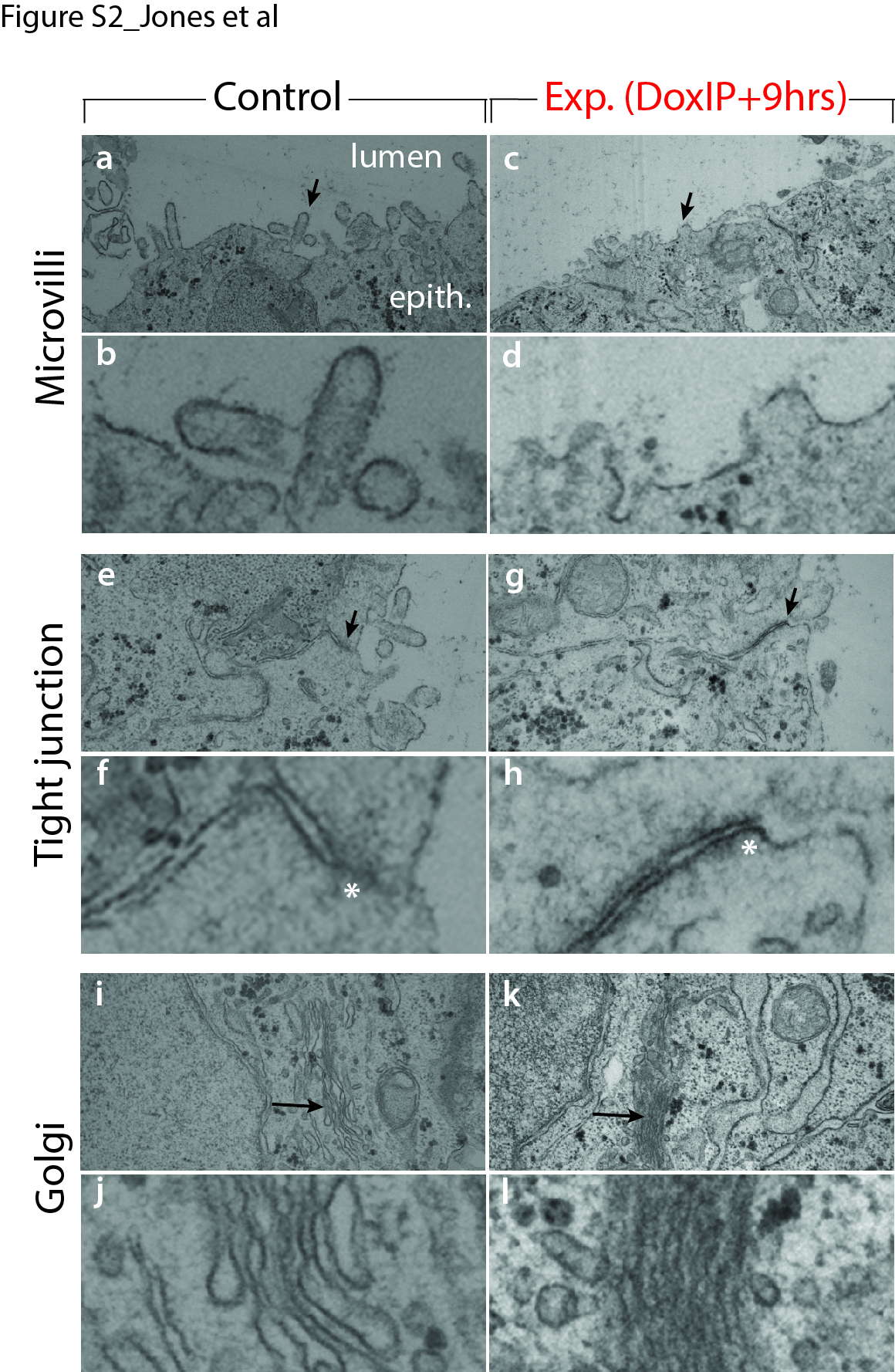

Supplement: Figure S2 — Transmission electron microscopy: Compared to controls, experimental lungs (DoxIP + 9 h) show reduced numbers and stunted microvilli (see black arrows, a–d), opened tight junctions (see black arrows in e and g; white asterisks in f and h), and flattened Golgi with increased staining (see black arrows, i–l). epith. = epithelium. Magnification: (a,c,e,g,i,k) 27,800x; (b,d,f,h,j,l) 139,000x. [file Image_2.JPEG]

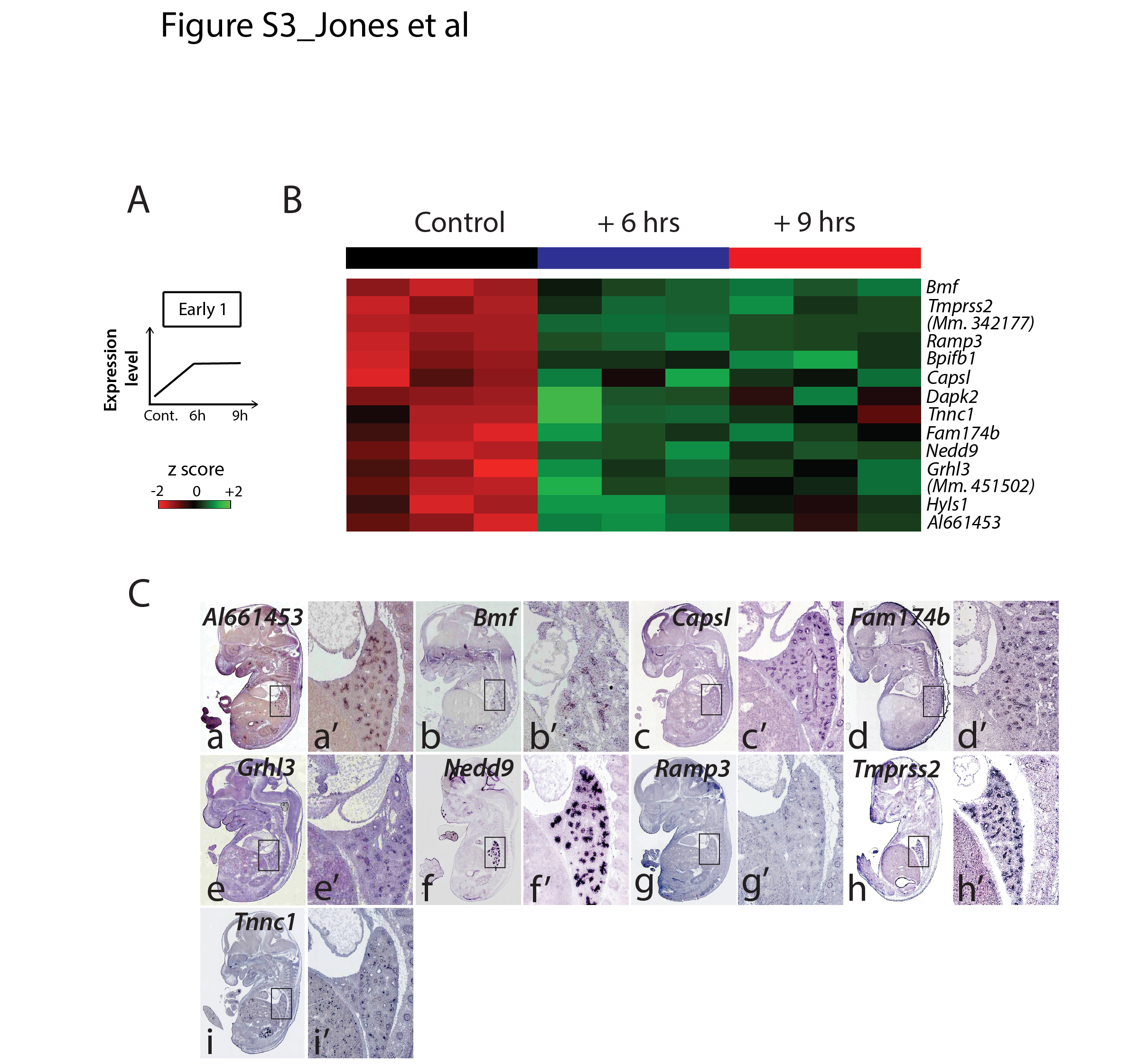

Supplement: Figure S3 — Genes and Expression pattern found in the Early 1 cluster (A) Graphical representation of changes in the level of gene expression over time. (B) Heat map. (C) Corresponding in situ hybridization results at E14.5 from genepaint. [file Image_3.JPEG]

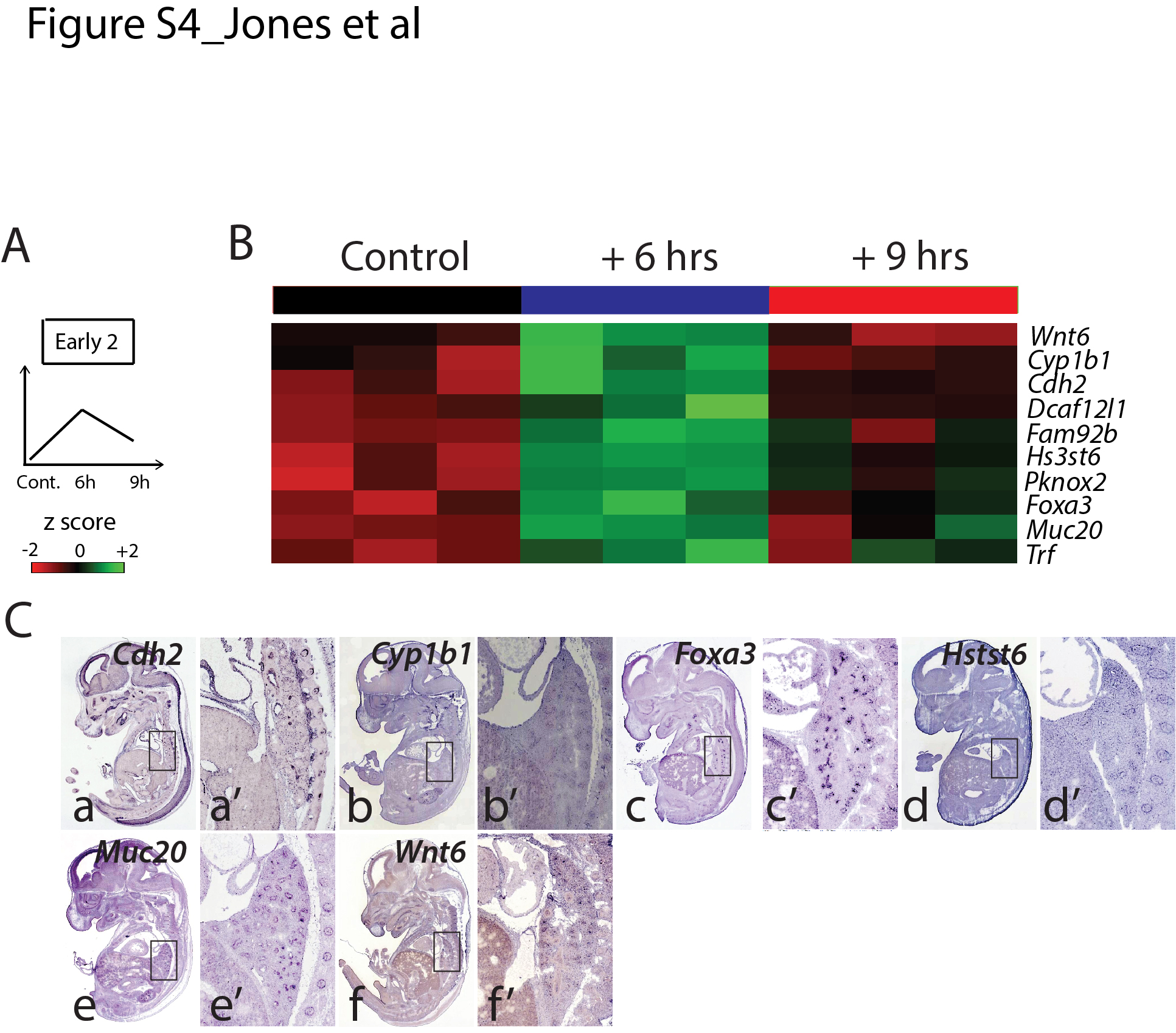

Supplement: Figure S4 — Genes and Expression pattern found in the Early 2 cluster (A) Graphical representation of changes in the level of gene expression over time. (B) Heat map. (C) Corresponding in situ hybridization results at E14.5 from genepaint. [file Image_4.JPEG]

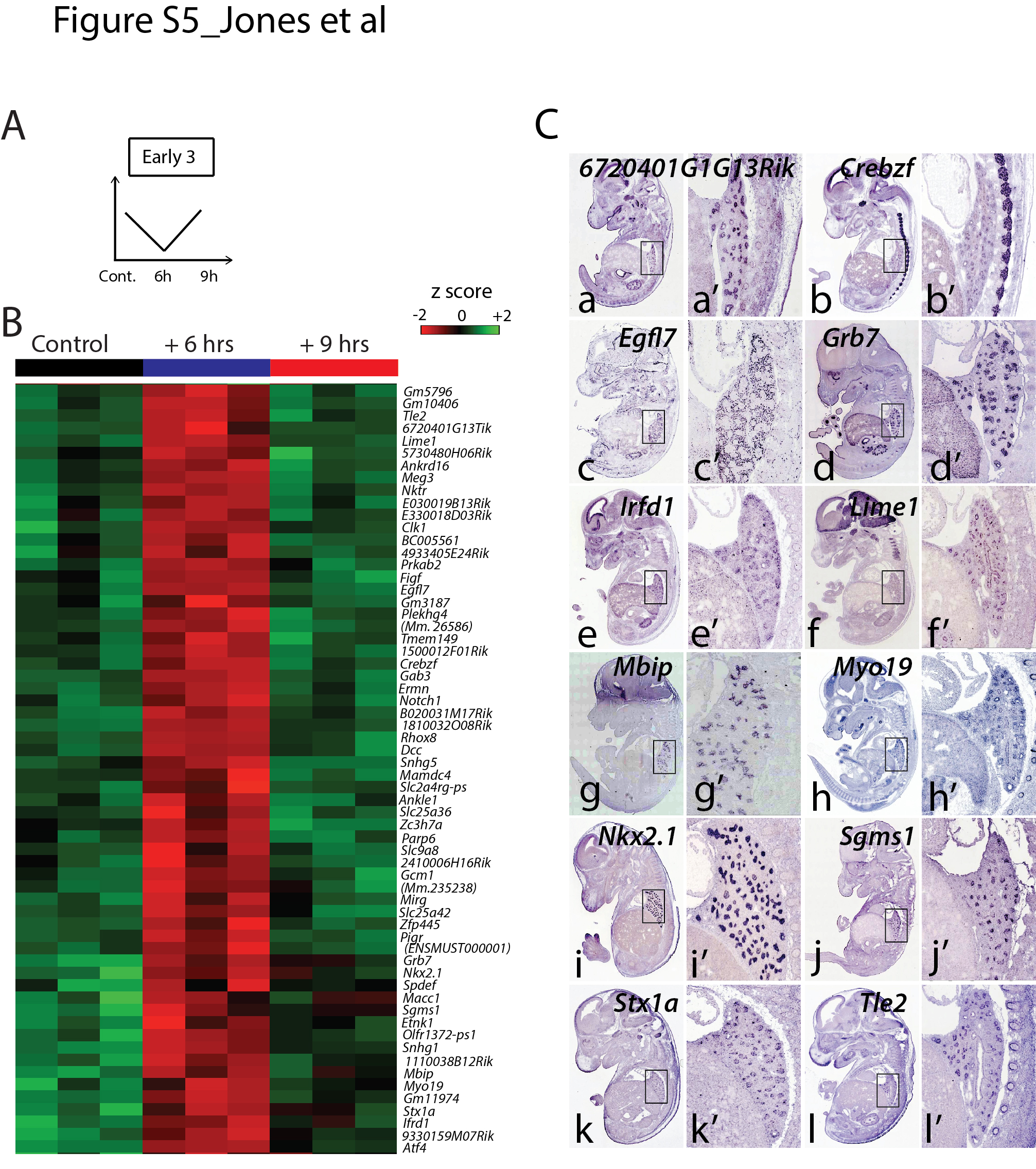

Supplement: Figure S5 — Genes and Expression pattern found in the Early 3 cluster (A) Graphical representation of changes in the level of gene expression over time. (B) Heat map. (C) Corresponding in situ hybridization results at E14.5 from genepaint. [file Image_5.JPEG]

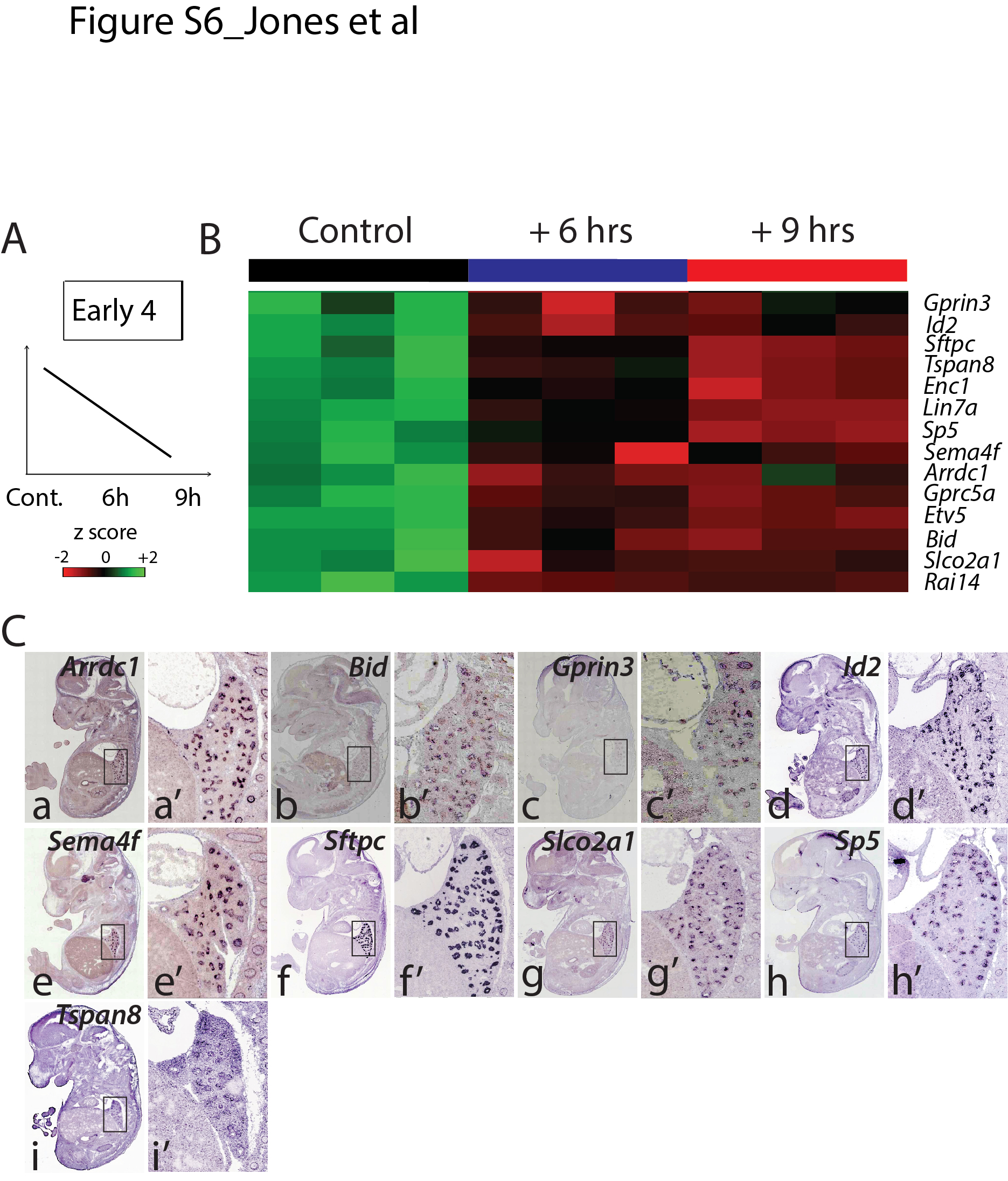

Supplement: Figure S6 — Genes and Expression pattern found in the Early 4 cluster (A) Graphical representation of changes in the level of gene expression over time. (B) Heat map. (C) Corresponding in situ hybridization results at E14.5 from genepaint. [file Image_6.JPEG]

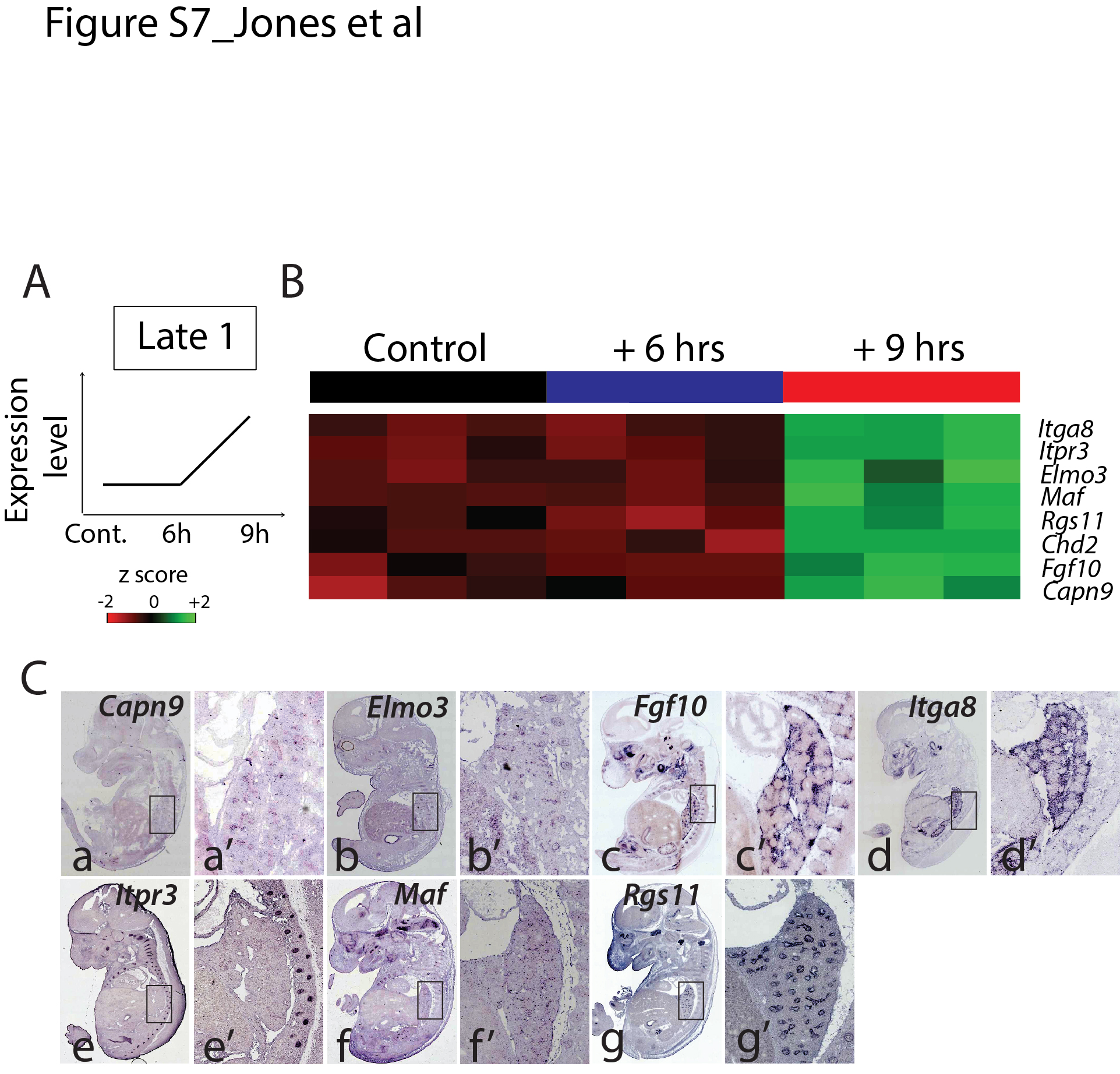

Supplement: Figure S7 — Genes and Expression pattern found in the Late 1 cluster (A) Graphical representation of changes in the level of gene expression over time. (B) Heat map. (C) Corresponding in situ hybridization results at E14.5 from genepaint. [file Image_7.JPEG]

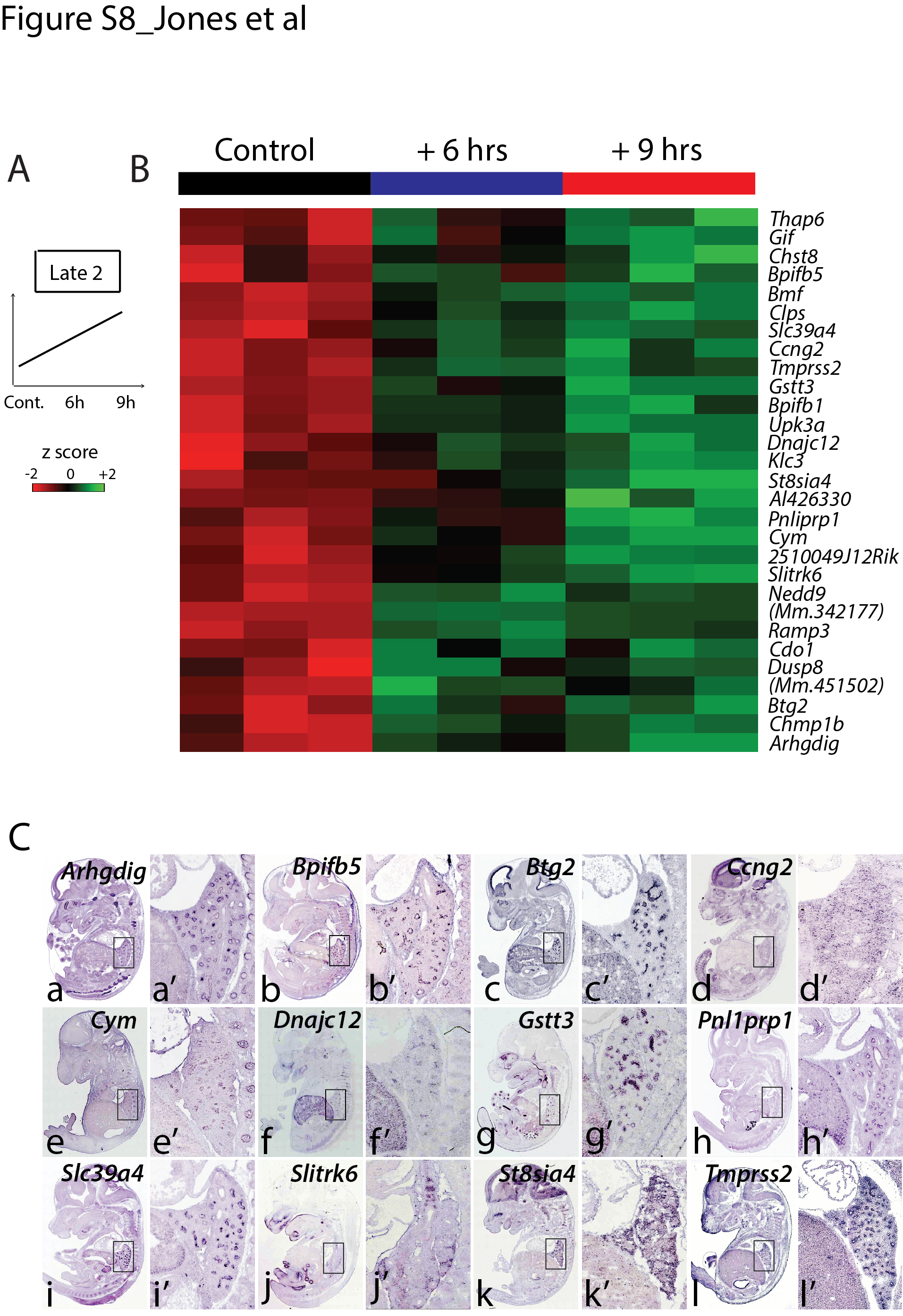

Supplement: Figure S8 — Genes and Expression pattern found in the Late 2 cluster (A) Graphical representation of changes in the level of gene expression over time. (B) Heat map. (C) Corresponding in situ hybridization results at E14.5 from genepaint. [file Image_8.JPEG]

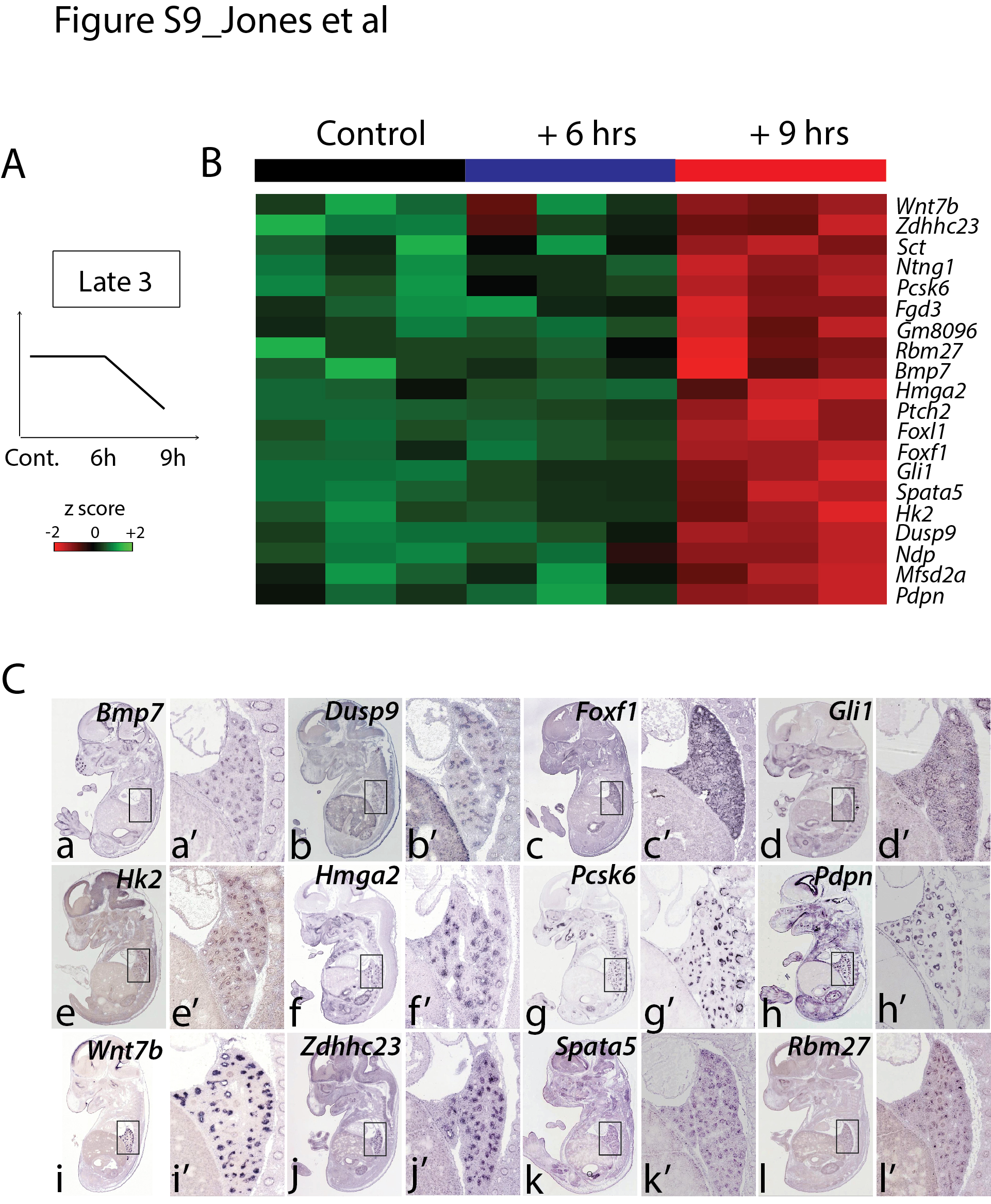

Supplement: Figure S9 — Genes and Expression pattern found in the Late 3 cluster (A) Graphical representation of changes in the level of gene expression over time. (B) Heat map. (C) Corresponding in situ hybridization results at E14.5 from genepaint. [file Image_9.JPEG]

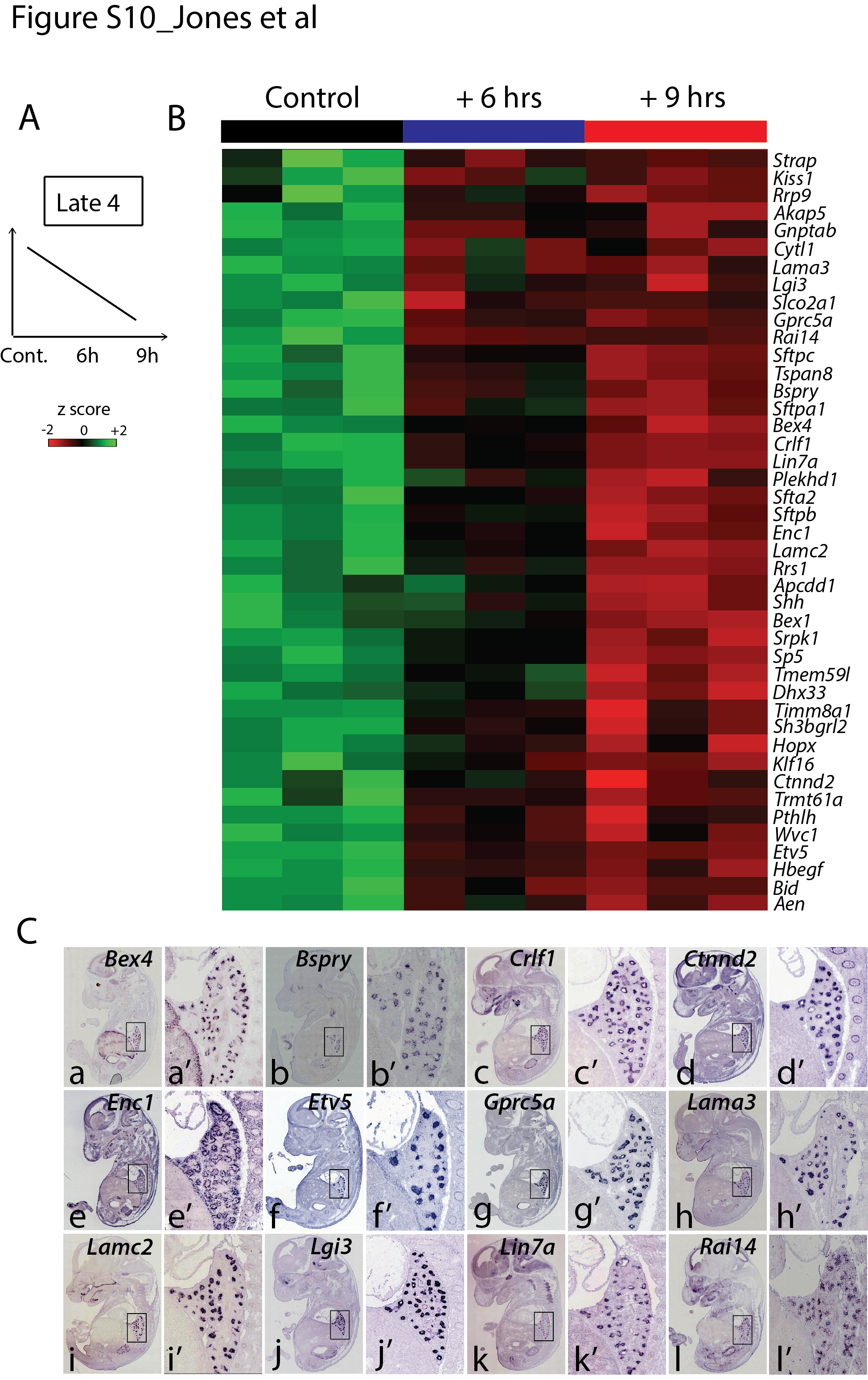

Supplement: Figure S10 — Genes and Expression pattern found in the Late 4 cluster (A) Graphical representation of changes in the level of gene expression over time. (B) Heat map. (C) Corresponding in situ hybridization results at E14.5 from genepaint. [file Image_10.JPEG]

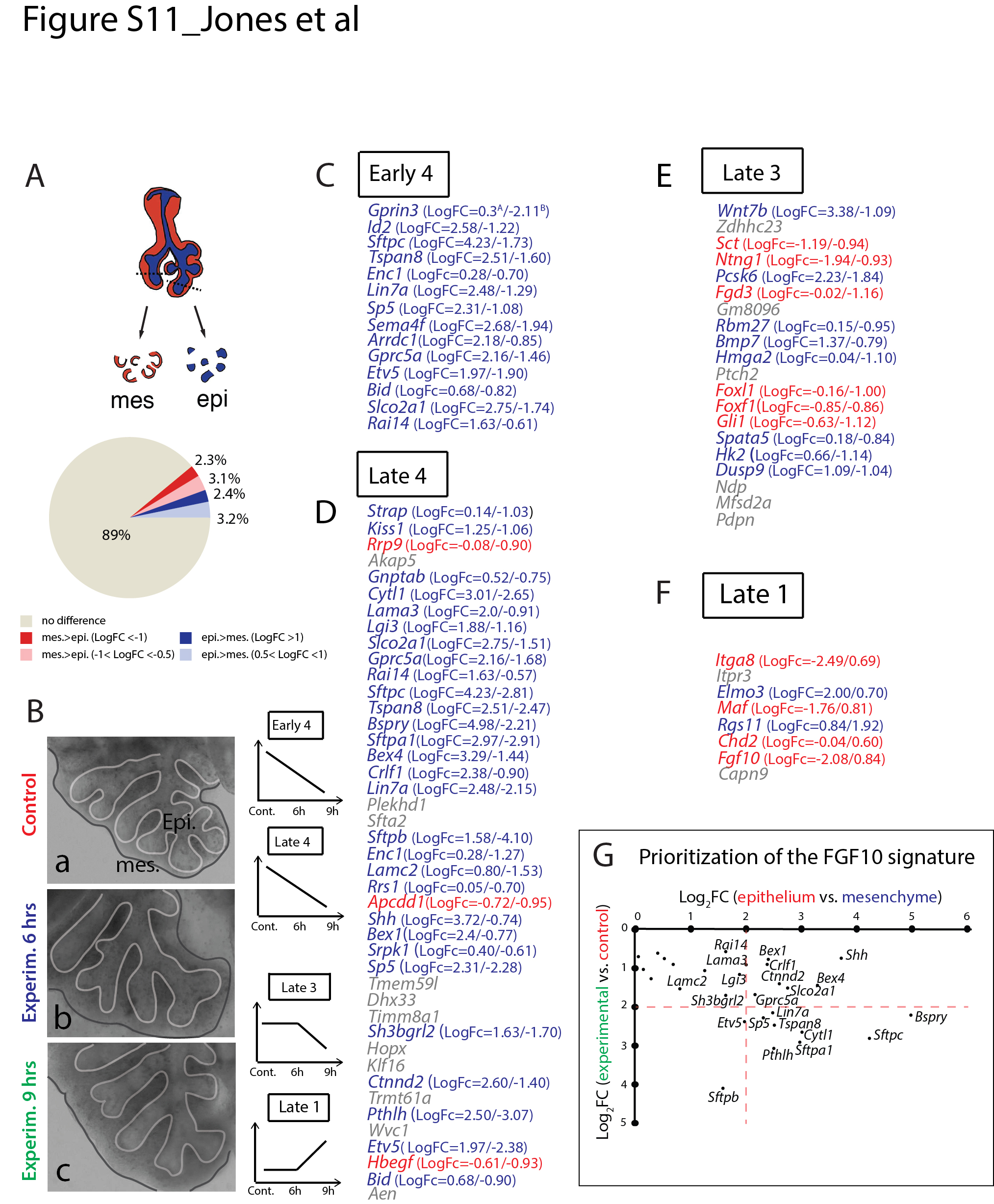

Supplement: Figure S11 — Relative level of expression of the genes of interest in epithelium and mesenchyme of WT E12.5 lungs and regulation of gene expression upon FGF10 inhibition (A) Determination of genes differentially expressed in the distal epithelium vs. mesenchyme of E12.5 wild type lungs by gene array (n = 3; see Materials and Methods for details on the statistical analysis of gene arrays). (B) Impact of in vivo FGF10 inhibition on lung branching at 6 and 9 h using our double transgenic system. Corresponding gene arrays allowed the identification of genes belonging to Early 4, Late 4, Late 3, and Late 1 groups. (C) Analysis of the genes found in the Early 4 cluster. The first LogFC (identified with “A” after the gene) represents the differential expression of this gene in the epithelium vs. mesenchyme of WT E12.5 lungs. The second LogFC (identified with “B” after the gene) represents the level of regulation upon FGF10 inhibition. Blue indicates genes enriched in the epithelium, and red indicates genes enriched in the mesenchyme. Genes in black were not found in our gene array in (A). Note that all the genes in Early 4 are blue, and therefore enriched in the epithelium. Some of these genes were differentially expressed in the epithelium at a high level (Log2FC more than 2; Id2, Sftpc, Tspan8, Lin7a, Sp5, Sema4f, Arrdc1, Gprc5a, and Slco2a1), a medial level (Log2FC between 1 and 2; Etv5 and Rai14), or a lower level (Log2FC between 0 and 1; Gprin3, Enc1, and Bid). We found that some of these genes were highly regulated upon FGF10 inhibition (Log2FC <-2; Gprin3), moderately regulated (Log2FC between −1 and −2; Id2, Sftpc, Tspan8, Lin7a, Sp5, Sema4f, Gprc5a, Etv5, Slco2a1), or weakly regulated (Log2FC between 0 and −1; Enc1, Arrdc1, Bid, Rai14). It is likely that genes which are expressed at high or medial levels in the epithelium, and which are highly or moderately regulated following inhibition of FGF10 activity, are involved in mediating FGF10 activity. One exception is Gprin3, whic [file Image_11.JPEG]
