# Supplementary material for: A Comprehensive Analysis of Fibroblast Growth Factor Receptor 2b Signaling on Epithelial Tip Progenitor Cells During Early Mouse Lung Branching Morphogenesis
Source: Front Genet. 2019 Jan 23;9:746. doi: 10.3389/fgene.2018.00746 (PMC6351499; doi:10.3389/fgene.2018.00746)
Supplement: Table S1 — Primary antibodies. [file Data_Sheet_1.docx]

**Supplementary tables**

**Table S1:** Primary antibodies

| **Antibody** | **Source** | **Identifier** |
| --- | --- | --- |
| Rabbit polyclonal anti-SOX2 | Novus Biologicals | Cat#NB 110-37235 |
| Rabbit polyclonal anti-SOX9 | Novus Biologicals | Cat#NBP 1-85551 |
| Rabbit polyclonal anti-Phospho-ßCatenin(Ser552) | Cell Signaling Technology | Cat#9566 |
| Rabbit monoclonal anti-LEF1 | Cell Signaling Technology | Cat#2230 |
| Mouse monoclonal anti-CDH1-FITC | BD Biosciences | Cat#612130 |
| Rabbit polyclonal anti-LAMA1 | Sigma-Aldrich | Cat#L9393 |

**Table S2:** Primer sequences for qRT-PCR

| **Primers** | **Forward** | **Reverse** | **Product size** |
| --- | --- | --- | --- |
| *Fgf10* | ATGACTGTTGACATCAGACTCCTT | CACTGTTCAGCCTTTTGAGGA | 75 bp |
| *Fgf1* | CTTTTATACGGCTCGCAGACAC | TTCTTGAGGCCCACAAACCA | 125 bp |
| *Fgf3* | CAAGCTCTACTGCGCTACCA | AGGATGCTATAGGCGCTGTT | 90 bp |
| *Fgf7* | ACTATCTGCTTATAAAATGGCTGCT | GTGGGGCTTGATCATCTGAC | 114 bp |
| *Ctnnd2* | CGACAACAAAAGCGGAGGAT | GGACAGGCATAGCTCCGAA | 167 bp |
| *Etv4* | AGGAGTACCATGACCCCCTG | GGACATCTGAGTCGTAGGCG | 138 bp |
| *Etv5* | TAGCTGAAGCACAAGTTCCTGA | GCAGCTCCCGTTTGATCTTG | 105 bp |
| *Sox9* | AGTCGGTGAAGAACGGACAA | CTGAGATTGCCCAGAGTGC | 158 bp |
| *Sftpa1* | CAGTGTGATTGGGAGAAACCA | ATGCCAGCAACAACAGTCAA | 88 bp |
| *Sftpb* | GGCTAGACAGGCAAAAGTGTG | GACCGCGTTCTCAGAGGTG | 171 bp |
| *Sftpc* | GGTCCTGATGGAGAGTCCAC | GATGAGAAGGCGTTTGAGGT | 94 bp |
| *Sp5* | CTGGCACACCAGGGTACTT | CCTCCTGGGGTAACTCAACT | 161 bp |
| *Shh* | TCACCCCCAATTACAACCCC | CTTGTCTTTGCACCTCTGAGTC | 89 bp |
| *Bspry* | GATTTTTGAGAGGGCCGAGGA | CCTTTCATCAATCAGCACCTCC | 166 bp |
| *Cytl1* | CGTGAGATCATGGCAGACTT | CCAGCACACAGTAGTTATGGA | 109 bp |
| *Pthlh* | ATTCCTACACAAGTCCCCAGA | AGCAGGAATACCAGGACACT | 84 bp |
| *Gprc5a* | TTGAGCGCTCTCGCCTTT | GTAGTCATTCTGGTTGGTCCTG | 87 bp |
| *Hprt* | TCCTCCTCAGACCGCTTTTT | ATCATCGCTAATCACGACGC | 82 bp |
